# Evidence human FTO catalyses hydroxylation of *N*^6^-methyladenosine without direct formation of a demethylated product contrasting with ALKBH5/2/3 and bacterial AlkB

**DOI:** 10.1093/nar/gkaf813

**Published:** 2025-08-28

**Authors:** Simranjeet Kaur, Pratheesh Maheswaran, Samanpreet Kaur, Yingqi Lai, Eidarus Salah, Dong Zhang, Shifali Shishodia, Christopher J Schofield, Wei Shen Aik

**Affiliations:** Department of Chemistry, Hong Kong Baptist University, Kowloon Tong, Hong Kong SAR, China; The Department of Chemistry and the Ineos Oxford Institute for Antimicrobial Research, Chemistry Research Laboratory, University of Oxford, 12 Mansfield Road, OxfordOX1 3TA, United Kingdom; The Department of Chemistry and the Ineos Oxford Institute for Antimicrobial Research, Chemistry Research Laboratory, University of Oxford, 12 Mansfield Road, OxfordOX1 3TA, United Kingdom; Department of Chemistry, Hong Kong Baptist University, Kowloon Tong, Hong Kong SAR, China; The Department of Chemistry and the Ineos Oxford Institute for Antimicrobial Research, Chemistry Research Laboratory, University of Oxford, 12 Mansfield Road, OxfordOX1 3TA, United Kingdom; The Department of Chemistry and the Ineos Oxford Institute for Antimicrobial Research, Chemistry Research Laboratory, University of Oxford, 12 Mansfield Road, OxfordOX1 3TA, United Kingdom; The Department of Chemistry and the Ineos Oxford Institute for Antimicrobial Research, Chemistry Research Laboratory, University of Oxford, 12 Mansfield Road, OxfordOX1 3TA, United Kingdom; The Department of Chemistry and the Ineos Oxford Institute for Antimicrobial Research, Chemistry Research Laboratory, University of Oxford, 12 Mansfield Road, OxfordOX1 3TA, United Kingdom; Department of Chemistry, Hong Kong Baptist University, Kowloon Tong, Hong Kong SAR, China

## Abstract

*N* ^6^-Methyladenosine (m^6^A) is a prevalent post-transcriptional modification in eukaryotic messenger RNA. Two cancer-linked human Fe(II) and 2-oxoglutarate (2OG)-dependent oxygenases, the fat mass and obesity associated-protein (FTO), and AlkB human homolog 5 (ALKBH5) catalyse m^6^A methyl group oxidation. While ALKBH5 has consistently been reported to catalyse m^6^A demethylation, there are conflicting reports concerning the FTO products. We report studies using mass spectrometry and nuclear magnetic resonance comparing products of FTO, ALKBH5, and DNA damage repair demethylases (human ALKBH2 and ALKBH3 and bacterial AlkB, using m^1^A single-stranded DNA substrates). The results with m^6^A-containing single-stranded RNA (ssRNA) and *N*^6^,2′-*O*-dimethyladenosine adjacent to the 5′ m^7^G triphosphate cap ssRNA substrates imply that the predominant FTO product is *N*^6^-hydroxymethyladenosine, either with or without methylation on the substrate ribose 2′-hydroxyl group. The nascent hemiaminal product undergoes relatively slow non-enzyme catalysed fragmentation giving adenosine/formaldehyde. The other four 2OG-dependent oxygenases tested, including ALKBH5, produce demethylated bases as the predominant products. The results imply that, at least in isolated form, FTO preferentially acts as a hydroxylase, producing a hemiaminal product, rather than a demethylase, distinguishing it from ALKBH5. They highlight a need for investigations into the roles of hemiaminal-type modifications to nucleic acids, in both healthy biology and disease.

## Graphical Abstract

**Graphical Abstract:**
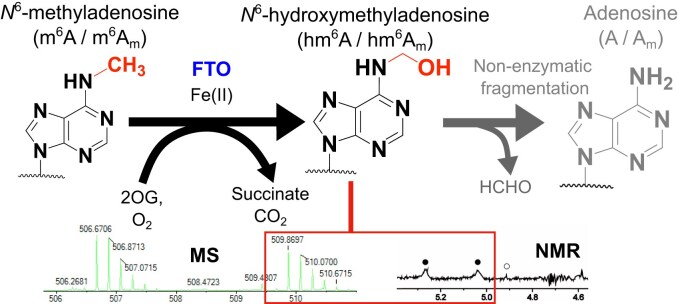

## Introduction

Methylation of messenger RNA (mRNA) is a common post-transcriptional modification that has roles in regulating mRNA function. Two important sites of mRNA methylation involve adenosine, i.e. internal *N*^6^-methyladenosine (m^6^A) and *N*^6^,2′-*O*-dimethyladenosine (m^6^A_m_), in the 5′ mRNA cap [1–5]. The internal m^6^A mark is located within the RR(m^6^A)CH consensus motif (R: G/A; H: A/C/U) [6, 7], while m^6^A_m_ modifications commonly occur in the nucleotide adjacent to the 5′ *N*^7^-methylguanosine (m^7^G) triphosphate cap [4, 8, 9].

Although multiple human *N*^ϵ^-methyl lysine histone demethylases (KDMs) have been identified [10–13], only two human oxygenases modifying the methyl group of m^6^A have been identified [14, 15], namely the fat mass and obesity associated-protein (FTO) [14, 16] and AlkB homolog 5 (ALKBH5) [15]. FTO and ALKBH5 are related by sequence and structure, and both are linked to cancer [17–22]; mutations of the *FTO* gene are also linked to other diseases, including obesity and brain disorders [23–26]. FTO is reported to catalyse demethylation of the 5′-cap m^6^A_m_ [27, 28]; ALKBH5, however, is reported to be inactive against m^6^A_m_ [29, 30]. Despite the biological importance of FTO and ALKBH5, as evidenced by cellular and genetic studies, how the reactions that they catalyse are connected to healthy development/physiology and disease is poorly understood.

**Figure 1.**
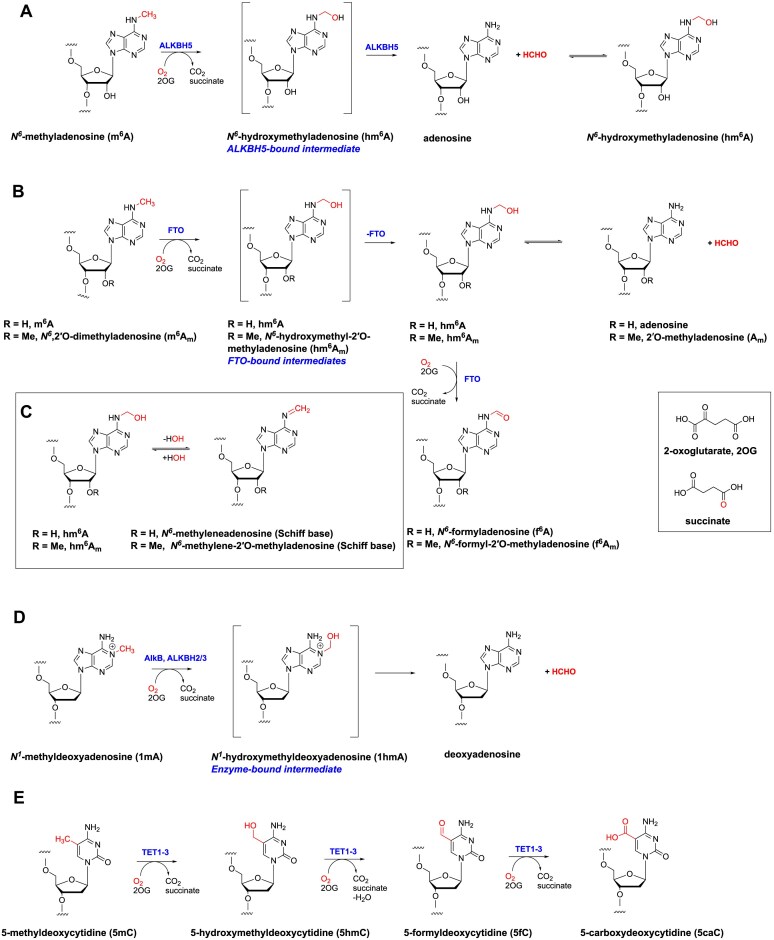
Reactions reported to be catalysed by ALKBH5 and FTO and related human 2OG-dependent oxygenases. Reactions catalysed by (**A**) ALKBH5, (**B**, **C**) FTO, (**D**) AlkB, ALKBH2/3, and (**E**) TET1-3 are shown. Note that ALKBH5 is proposed to catalyse demethylation via a transient enzyme-bound *N*^6^-hydroxymethyladenosine (hm^6^A) intermediate. In the case of FTO, in addition to demethylated adenosine, evidence has been reported for additional products, i.e. hm^6^A, an imine, and f^6^A. Note that tautomeric states other than those shown may be relevant.

Both FTO and ALKBH5 are Fe(II) and 2-oxoglutarate (2OG)-dependent oxygenases that employ 2OG and O_2_ as co-substrates to oxidize m^6^A, producing succinate, CO_2_, and formaldehyde (HCHO) as co-products [16, 31–34]. There are 60–70 human 2OG oxygenases [34–36], which, *inter alia*, play important roles in collagen biosynthesis [37], lipid metabolism [38], epigenetic regulation [39, 40], the hypoxic response [41], and DNA damage repair [40, 42]. In the latter case, human homologues (ALKBH2, ALKBH3) of bacterial AlkB are reported to catalyse damage repair via oxidation of DNA-damaging alkylations, resulting in fragmentation to give an aldehyde and the repaired base (Fig. 1D) [43–46]. Some 2OG oxygenases (e.g. AlkB [40, 42, 44, 47]) manifest broad substrate and product selectivities, but this is not always the case [31].

Interestingly, there is evidence that FTO and ALKBH5 produce different products with the same m^6^A-containing substrates [33, 48, 49]. When reacted with m^6^A-containing ssRNA in the presence of Fe(II) and 2OG, FTO has been reported to produce not only *N*^6^-demethylated adenosine but also hm^6^A, *N*^6^-formyladenosine (f^6^A), and *N*^6^-methyleneadenosine (imine) [32, 33, 49]; by contrast, ALKBH5 has been reported to produce only *N*^6^-demethylated adenosine [33, 48, 50] (Fig. 1A–C). Crystal structures of ALKBH5–m^6^A RNA complexes have led to a proposed mechanism for ALKBH5-catalysed hm^6^A fragmentation, which is promoted by a proton shuttling machinery involving Lys132_ALKBH5_ and Tyr139_ALKBH5_, a mechanism not apparent in the FTO active site [48, 50].

Studies on the reactions of HCHO with canonical and modified nucleobases have shown that HCHO reacts with endocyclic nitrogens to give equivalent hemiaminal products, as observed with thymidine and uridine monophosphates [49]. Such endocyclic reactions of HCHO are faster than with exocyclic nitrogens; however, the exocyclic hemiaminal adducts, as formed with guanine, cytosine, and, in particular, adenine, are more stable in solution [49]. FTO-catalysed hydroxylation of the m^6^A nucleoside (m^6^A [^13^C]-labelled on its 6-methyl group) was shown to give hm^6^A as the major product as observed by nuclear magnetic resonance (NMR); by contrast, (3-methyl)thymidine was observed to undergo demethylation, consistent with the decreased stability of the likely endocyclic hemiaminal intermediate in the case of (3-methyl)thymidine demethylation compared to the analogous exocyclic hemiaminals [49]; this work did not describe comparative studies with ALKBH5 [49, 51].

Some reported methods used to analyse FTO products/mRNA modification might not preserve hm^6^A/hm^6^A_m_ adducts and other hemiaminal-type covalent modifications that are relatively unstable in aqueous environments [14, 32, 33, 52–54]. Here, we report studies using high-resolution mass spectrometry (MS) and real-time NMR to analyse modifications to m^6^A-containing ssRNA produced as a consequence of FTO and ALKBH5 catalysis, comparing the results with those for the apparent *bona fide* demethylases ALKBH2, ALKBH3, and bacterial AlkB. The combined results clearly demonstrate that, in contrast to the other tested oxygenases, isolated FTO preferentially acts as a hydroxylase rather than a demethylase, highlighting a need for further investigations into the biological roles of hm^6^A and hm^6^A_m_.

## Materials and methods

### Production and purification of FTO, FTOΔ31, ALKBH5_66–292_, ALKBH5_74–292_, ALKBH5_74–292_ K132E, AlkBΔN11, ALKBH2, and ALKBH3 recombinant proteins

Recombinant forms of FTO [54], FTOΔ31 [48, 55], ALKBH5_66–292_ [56], ALKBH5_74–292_ [48], and ALKBH5_74–292_ K132E [48] were produced as previously reported. DNA constructs containing AlkBΔN11, full-length ALKBH2, and full-length ALKBH3 were cloned into the pET-28a(+) vector using the NcoI and BamHI, NdeI and NcoI, and NcoI and HindIII restriction sites, respectively. In brief, *Escherichia coli* BL21 (DE3) cells were transformed with the respective expression vectors and grown in LB medium at 37°C with shaking until the culture reached an optical density at 600 nm (OD_600_) of 0.6–0.8. Protein production was induced by the addition of isopropyl β-d-1-thiogalactopyranoside (IPTG) to a final concentration of 0.5 mM. The cell cultures were incubated at 18°C with shaking at 150–180 rpm for ∼18 h. The cells were harvested by centrifugation (8000 rpm, 10 min, 4°C); the resulting pellets were stored at −80°C. Frozen cell pellets were resuspended in lysis buffer (20 mM Tris–HCl, pH 7.5, 500 mM NaCl, and 10 mM imidazole) supplemented with 1 mg DNase I and a cOmplete™ protease inhibitor cocktail (Roche) and lysed by sonication on ice. The lysates were then cleared by centrifugation (20 000 rpm for 30 min, 4°C), and the supernatant was loaded onto a 5-ml HisTrap HP column (GE Healthcare) pre-equilibrated with binding buffer (20 mM Tris–HCl, pH 7.5, 500 mM NaCl, and 10 mM imidazole). The column was washed with wash buffer (20 mM Tris–HCl, pH 7.5, 500 mM NaCl, and 40 mM imidazole) and eluted with elution buffer (20 mM Tris–HCl, pH 7.5, 500 mM NaCl, and 500 mM imidazole). The eluted protein was further purified using a Superdex 75 300-ml column (Cytiva) pre-equilibrated with buffer containing 50 mM Tris–HCl (pH 7.5) and 150 mM NaCl.

### Quadrupole Time-of-Flight Mass Spectrometry (QTOF-MS)-based *in vitro* m^6^A_m_ and m^6^A hydroxylation assays

Reaction mixtures containing 0.5 μM FTOΔ31, 0.5 μM ALKBH5_74–292_ or ALKBH5_74–292_ K132E, 20 μM m^7^Gpppm^6^A_m_UACUU ssRNA substrate (Biosynthesis, USA) or UGGm^6^ACUGC ssRNA (Horizon Discovery), 200 μM 2OG disodium salt, 100 μM diammonium Fe(II) sulfate, 1 mM sodium l-ascorbate, and with or without 25 μM 2,4-PDCA inhibitor (for ALKBH5_74–292_ and ALKBH5_74–292_ K132E) in 25 mM Tris (pH 7.2) were prepared and incubated at 37°C. Reactions were sampled at various time points between 0 and 45 min, then purified using C-18 Ziptips (Thermo Fisher) (purification time = 5 min), and immediately snap-frozen and stored in liquid nitrogen. The samples were later thawed and subjected to QTOF-MS analysis (SCIEX Triple TOF 6600). For data acquisition, the sample [in 50% (v/v) MeCN] was injected directly into the spectrometer at 30 μl/min. One hundred to one hundred twenty scans were accumulated in the negative mode, and total ion count was used to quantify the signal ion intensities. Data analysis was performed by the Sciex OS Explorer software. The plots were generated using GraphPad Prism 10.1.1.

### QTOF-MS-based hm^6^A_m_ and hm^6^A decay assays

Initial hydroxylation reactions were initiated by preparing reaction mixtures as described above with incubation at 37°C for 10 min (hm^6^A_m_) or 30 min (hm^6^A). The mixture was then divided equally. A final concentration of 1 mM FTO inhibitor 2,4-PDCA [55–57] was added to one of the portions, 1 mM; an equivalent volume of reaction buffer was added to the other portion. The samples were incubated at 37°C and sampled every 45 min until 450 min. They were then purified using C-18 Ziptips (Thermo Fisher) (purification time = 5 min) and immediately snap-frozen and stored in liquid nitrogen. The samples were later thawed and subjected to QTOF-MS analysis (SCIEX Triple TOF 6600). Procedures for data acquisition, processing, and analysis were as described for the QTOF-MS-based *in vitro* m^6^A_m_ and m^6^A hydroxylation assays. Data analyses were performed using GraphPad Prism 10.

### Ion-pairing reversed-phase liquid chromatography–mass spectrometry time-course assays

Solutions containing His_6_-tagged enzyme [FTO (0.25 or 0.5 μM), ALKBH5 (2 or 4 or 8 μM), or AlkBΔN11 (0.2 μM)/ALKBH2 (0.5 μM)/ALKBH3 (0.5 μM)], 100 μM sodium-l-ascorbate (500 μM for ALKBH5), 10 μM ammonium iron(II) sulfate hydrate (50 μM for ALKBH5), and 10 μM 2OG disodium salt (50 μM for ALKBH5) was added to 20 μM of substrate in 50 mM HEPES buffer and 0.1% (v/v) Tween 20 (pH 7.5) (230 μl final reaction volume). 30 μl of the mixture extract was taken at each time point and quenched by adding 60 μl of 1.33% (v/v) aqueous acetic acid for LC/MS analysis. Samples were transferred to an Eppendorf vial, then frozen using liquid nitrogen and stored at −20°C. Prior to MS analysis, samples were thawed and centrifuged (14 000 rpm, 20 min, 4°C); 10 μl was used for analysis. UGGm^6^ACUGC (ssRNA, 8-mer) was obtained from Dharmacon custom RNA synthesis (Horizon Discovery). AAAGCAGm^1^AAATTCGAAAAAGCGAA (ssDNA, 24-mer) and CAm^1^AAT (ssDNA, 5-mer) were obtained from Keck Oligos. The dried oligonucleotides were reconstituted to 1 mM and diluted to 200 μM in UltraPure DNase/RNase-free distilled water.

A Waters^®^ ACQUITY UPLC Oligonucleotide BEH C18 Column (130 Å, 1.7 μm, 2.1 mm × 50 mm) with a gradient of 98% (v/v) buffer A to 70% (v/v) buffer B over 8 min at room temperature was used for purification. Buffer A: 200 mM HFIP, 8.15 mM Triethylamine (TEA) buffer, and 5% (v/v) methanol. Buffer B: 20% (v/v) buffer A + 80% (v/v) methanol. The LC/MS system was operated using MassLynx™ version 4.1 (Waters Corp., Milford, MA, USA). LC/MS chromatograms were acquired in the negative ion full scan mode using an ESI-MS capillary voltage of 2.5–3.0 kV, a sample cone voltage of 40 V, and an MCP detector voltage of 3000 V. The desolvation gas flow rate was 800 l/h. The cone gas flow rate was set to 30 l/h. The desolvation temperature and source temperature were set to 400°C and 150°C, respectively. Relevant molecular ions were extracted, and the reaction progress curve was plotted using GraphPad Prism 5.

### NMR monitoring of FTO and ALKBH5 using ^13^C-labelled m^6^A nucleoside as substrate

The ^13^C-m^6^A nucleoside with *N*^6^-methyl group selectively labelled with ^13^C was synthesized as reported [49]. The FTO-catalysed reaction was monitored using both ^1^H NMR and the gradient-selected 1D heteronuclear single quantum correlation (^1^H–^13^C-HSQC) method. In brief, samples containing FTO or ALKBH5 (20 μM, final concentration, final reaction volume: 160 μl; enzymes were stored in Tris buffer), ^13^C-m^6^A (400 μM), 2OG (5 mM), sodium l-ascorbate (1 mM), and Fe(II) ammonium sulfate (20 μM) in ammonium formate buffer in D_2_O (pD 7.9) were mixed and immediately transferred to a 3-mm-diameter MATCH NMR tube (Hilgenberg). The time lag between sample mixing and the first acquisition completion was 15 min. Spectra were acquired using 1D NOESY water pre-saturation (Bruker pulse program: *noesygppr1d*) using 160 scans and a relaxation delay of 2 s, and 1D ^1^H–^13^C-HSQC (Bruker pulse program: hsqcgpclip1d) using 400 scans with a relaxation delay of 2 s. The HSQC was optimized for a ^1^*J*_CH_ coupling of 145 Hz. NMR spectra were measured using a Bruker AVIII 700 MHz NMR spectrometer equipped with a TCI helium cooled cryoprobe. Data were processed with TopSpin v.3.5.6. The temperature of the probe was 310 K. The time course was run for 720 min.

### HPLC-based m^6^A nucleoside hydroxylation assays

Reaction mixtures containing 10 μM FTO, 100 μM *N*^6^-methyladenosine (m^6^A) nucleoside substrate, 200 μM 2OG disodium salt, 100 μM diammonium Fe(II) sulfate, and 1 mM sodium l-ascorbate in 25 mM Tris (pH 7.2) were prepared in a final volume of 50 μl and incubated at 37°C for 5 min. Reactions were quenched by heating the mixture at 90 °C for 5 min. Fifty microlitres of methanol was then added, and the mixtures were centrifuged to remove the precipitated protein. The mixtures were injected into an Agilent Technologies 1200 Infinity high-performance liquid chromatography (HPLC) equipped with a UV detector. The product (adenosine) and substrate (m^6^A) were separated using an Agilent 5 HC C18(2), 250 mm × 4.6 mm column, at a flow rate of 0.5 ml/min. The UV detector was set at a wavelength of 254 nm. The mobile phase consisted of H_2_O with 0.1% trifluoroacetic acid (TFA) as buffer A and acetonitrile with 0.1% TFA as buffer B. The gradient program was as follows: 0–20 min, 2%–4% B; 20–40 min, 4%–8% B; 40–41 min, 8%–100% B; and 41–55 min, 100% B. UV peaks were integrated using MestReNova 14.2.3. For the determination of the *K*_M_ of m^6^A nucleoside, assays were conducted in triplicate across a range of substrate concentrations (80 μM, 160 μM, 320 μM, 640 μM, 1.28 mM, 2.56 mM, 5.12 mM, and 10.24 mM). The *K*_M_ value was calculated from the Michaelis–Menten model using GraphPad Prism 10.1.1.

### NMR monitoring of FTO and ALKBH5 using a 5-mer m^6^A-containing ssRNA as substrate

m^6^A phosphoramidite and GGm^6^ACU were synthesized as reported [58, 59]. Reactions contained FTO or ALKBH5 (20 μM), ssRNA GGm^6^ACU (200 μM), 2OG (1 mM), l-ascorbate (1 mM), Fe(II) ammonium sulfate (80 μM), and 1 ml of 1 mg/ml TSP as an internal standard in ammonium formate buffer in D_2_O (pD 7.9). The first acquisition was completed 15 min after addition. Spectra were acquired using 1D NOESY water pre-saturation (Bruker pulse program: *noesygppr1d*) using 16 (ALKBH5) or 32 (FTO) scans and a relaxation delay of 2 s. Control experiments’ spectra (ssRNA and HCHO spiked in) were acquired for 160 scans. The temperature of the probe was 310 K for the reaction with FTO. For the reaction of ALKBH5, the temperature of the probe was 298 K for the first 25 min, and then 310 K for the remainder of the time course. NMR spectra were measured using a Bruker AVIII 700 MHz NMR spectrometer equipped with a TCI helium cooled cryoprobe. Data were processed with TopSpin v.3.5.6. The samples were prepared in 3-mm-diameter MATCH NMR tubes (Hilgenberg).

### NMR monitoring of FTO and ALKBH5 on unlabelled m^6^A nucleoside or 8-mer m^6^A-containing ssRNA as substrate

Assay mixtures contained ALKBH5 (20 μM) or full-length FTO (10 μM); for reactions with ssRNA UGGm^6^AACUGC (800 μM), full-length FTO (20 μM) or ALKBH5 (20 μM) was used. For reactions in the absence of substrate, full-length FTO (20 μM) was used. m^6^A nucleoside was purchased from Sigma–Aldrich and ssRNA UGGm^6^AACUGC was purchased from Dharmacon RNA synthesis. With the m^6^A nucleoside potential substrate, reactions contained FTO or ALKBH5 (20 μM), 2OG (800 μM), Fe(II) ammonium sulfate (200 μM), m^6^A nucleoside (800 μM), sodium l-ascorbate (1 mM), and an internal standard (TSP, 800 μM). For FTO reactions, 50 mM ammonium formate buffer in D_2_O (pD 7.5) was used; for ALKBH5 reactions, 50 mM ammonium formate buffer in D_2_O (pD 7.9) was used. For the reaction of FTO with ssRNA UGGm^6^AACUGC, 2OG (5 mM) and Fe(II) ammonium sulfate (400 μM) were used. All other concentrations were the same.

NMR spectra were measured using a Bruker AVIII 700 MHz NMR spectrometer equipped with a TCI helium cooled cryoprobe. Data were processed with TopSpin v.3.6.2. 1D NOESY with pre-saturation (Bruker pulse program: *noesygppr1d*) using 64 scans and a relaxation delay of 2 s; data were treated with an exponential function with 2 Hz line broadening, prior to Fourier transformation, with the exception of the full-length FTO reaction without substrate where 0.3 Hz line broadening was applied, due to the low intensity of the succinate peak. Spectra were acquired every 5 min over 120 min. For the reaction of full-length FTO with ssRNA UGGm^6^ACUGC, two additional time points were obtained, i.e. at ∼2880 min, before and after the addition of H^13^CHO. One additional time point was acquired at ∼2880 min for the uncoupled reaction of full-length FTO in the absence of substrate. All spectra were recorded at 298 K. All samples were prepared in 3-mm-diameter MATCH NMR tubes (CortecNet).

## Results

### FTO catalyses internal m^6^A hydroxylation and ALKBH5 catalyses m^6^A demethylation in ssRNA

**Figure 2.**
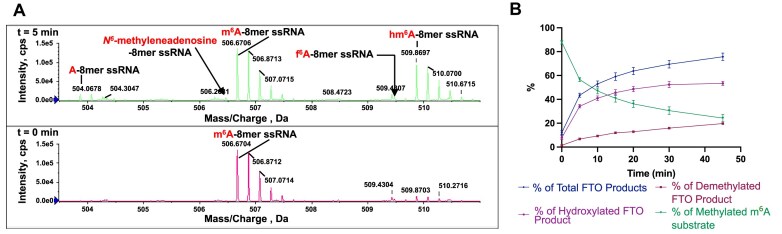
Evidence FTO acts as hydroxylase and ALKBH5 as a demethylase. (**A**) QTOF-MS time-course analysis of internal 8-mer m^6^A ssRNA (UGGm^6^ACUGC) by FTOΔ31. The extracted spectra at different time points indicate the 8-mer m^6^A ssRNA (*m*/*z* 506.67, −5 charge state) is converted to a hydroxylated product (hm^6^A) (*m*/*z* 509.86, −5), which was the major product throughout the time course (45 min). Peaks for the *N*^6^-demethylated product (*m*/*z* 503.86, −5) increase as the reaction progresses, likely due to non-enzymatic fragmentation of hm^6^A. Evidence for formation of f^6^A (*m*/*z* 509.46, −5) and imine products (*m*/*z* 506.46, −5) (indicated by arrows) was not accrued under these conditions. (**B**) Plots showing the reaction product profile of internal 8-mer m^6^A ssRNA oligo by FTOΔ31 at different time points (prepared using GraphPad Prism 10.1.1). Reactions were performed in triplicate (*n* = 3); error bars represent the standard error of the mean (SEM) (see also Supplementary Fig. S3).

To compare the activities of recombinant FTO and ALKBH5, we initially produced the proteins in highly purified forms via reported procedures [48, 54–56]. For FTO, we used both full-length FTO and a truncated FTO construct (FTO_32–505,_ FTOΔ31); the truncated construct has been shown to have similar activity to full-length FTO [60]. We then analysed their reactions, using an 8-mer m^6^A-containing ssRNA substrate (8-mer m^6^A ssRNA) with m^6^A at the fourth position (UGGm^6^ACUGC) (Fig. 2). Incubations were carried out as reported in the presence of Fe(II), O_2_, 2OG, and ascorbate at pH 7.2 or 7.5 [48, 49].

We initially monitored full-length FTO (0.5 and 0.25 μM)-catalysed substrate depletion and product formation (pH 7.5) over 45 min using ion-paired chromatography followed by negative ion electrospray ionization (ESI)-MS (IP-RP-LC/ESI-MS) using a XEVO G2-QTOF spectrometer with the same 8-mer m^6^A RNA substrate (Supplementary Figs S1A and S2). The major observed product of FTO catalysis was hm^6^A (1276.1832, corresponding to the −2 charge state), with the *N*^6^-demethylated product comprising <5% of the total products; there was no evidence for f^6^A formation. A low-level peak potentially corresponding to an *N*^6^-methyleneadenosine/imine (or other tautomer) was also observed (1267.6827, −2). By contrast, analysis of ALKBH5_66–292_ catalysis at various concentrations (8, 4, and 2 μM) using the same method showed that the major product was the *N*^6^-demethylated species (*m*/*z* 1261.1696, −2), with only very low levels of a peak corresponding to hm^6^A formation (1276.0139, −2) being observed, possibly reflecting reaction of HCHO produced by demethylation with the demethylated product (Supplementary Figs S1B and S3).

We then monitored FTOΔ31-catalysed substrate depletion and product formation at pH 7.2, that in the cell nucleus, over 45 min by direct injection into a SCIEX Triple TOF 6600 (QTOF) mass spectrometer, analysing in the negative ion mode (Fig. 2 and Supplementary Fig. S4). At early time points (<5 min), we observed a new peak corresponding to hm^6^A (*m*/*z* 509.86, −5) and 8-mer m^6^A ssRNA substrate (*m*/*z* 506.67, −5), with no evidence for *N*^6^-demethylated 8-mer ssRNA (*m*/*z* 503.86, −5) (Fig. 2A and Supplementary Fig. S4). A peak corresponding to *N*^6^-demethylated 8-mer ssRNA was first observed after 5 min; the intensity of this peak increased over the subsequent time course. Notably, at all the time points up to 45 min, hm^6^A was the dominant observed product (Fig. 2B and Supplementary Fig. S4). Low levels of peaks potentially corresponding to f^6^A (*m*/*z* 509.46, −5) and an imine (*m*/*z* 506.46, −5) were also observed over the course of the reaction, but the intensities of these peaks were insufficient to assign them as enzyme products; note that evidence for imine was not observed by ^1^H NMR (see below) and it is possible that conversion of m^6^A to the imine occurs in the mass spectrometer [49].

**Figure 3.**
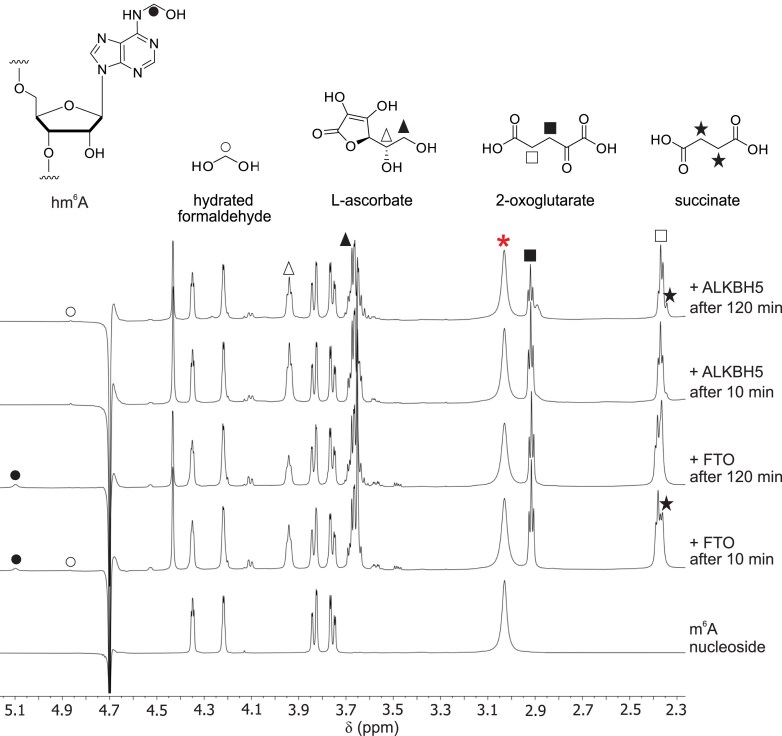
NMR evidence that FTO is a hydroxylase using m^6^A nucleoside as a substrate, while ALKBH5 is unable to efficiently catalyse m^6^A nucleoside oxidation. ^1^H NMR time-course spectra (700 MHz) of m^6^A nucleoside oxidation as catalysed by full-length FTO and potential m^6^A nucleoside oxidation by ALKBH5 are shown. FTO mediated oxidation is observed after 120 min as evidenced by a decrease in peak area of the broad resonance at *δ*_H_ 3.02 ppm (red asterisk) corresponding to m^6^A *N*^6^-CH_3_; this resonance was unchanged with ALKBH5. The peak at *δ*_H_ 5.10 ppm (black circle) corresponding to hm^6^A hemiaminal increased with FTO; no evidence for the hemiaminal was observed with ALKBH5. With FTO, there was a decrease in the 2OG methylene peaks (*δ*_H_ 2.93 ppm, black squares; 2.37 ppm, open squares) correlating with an increase in the succinate peak (*δ*_H_ 2.34 ppm, black star). With ALKBH5 there was only a slight decrease in the 2OG peak and a slight increase in the succinate peaks indicating substrate uncoupled or non-enzymatic oxidation of 2OG. Low levels of hydrated formaldehyde (*δ*_H_ 4.87 ppm, open circles) were observed with FTO and ALKBH5, likely substantially derived from the ammonium formate buffer.

We then analysed the FTO and ALKBH5 reactions by ^1^H NMR (700 MHz), initially at 25°C using an m^6^A nucleoside substrate. Consistent with prior NMR studies [49], full-length FTO catalysed oxidation of the m^6^A nucleoside to give the hm^6^A product, as observed by appearance of a broad peak corresponding to a hemiaminal methylene at *δ*_H_ 5.1 ppm (Fig. 3). Clear evidence for conversion of 2OG to succinate was observed by ^1^H NMR, but at a higher level than for conversion of m^6^A to hm^6^A, potentially reflecting substrate analogue promoted uncoupled 2OG turnover (Fig. 3 and Supplementary Figs S5 and S6). By contrast, there was no evidence for ALKBH5-catalysed oxidation of the m^6^A nucleoside and only low levels of turnover of 2OG to succinate were observed (Supplementary Fig. S7), possibly reflecting uncoupled and/or non-enzymatic reaction [61, 62].

**Figure 4.**
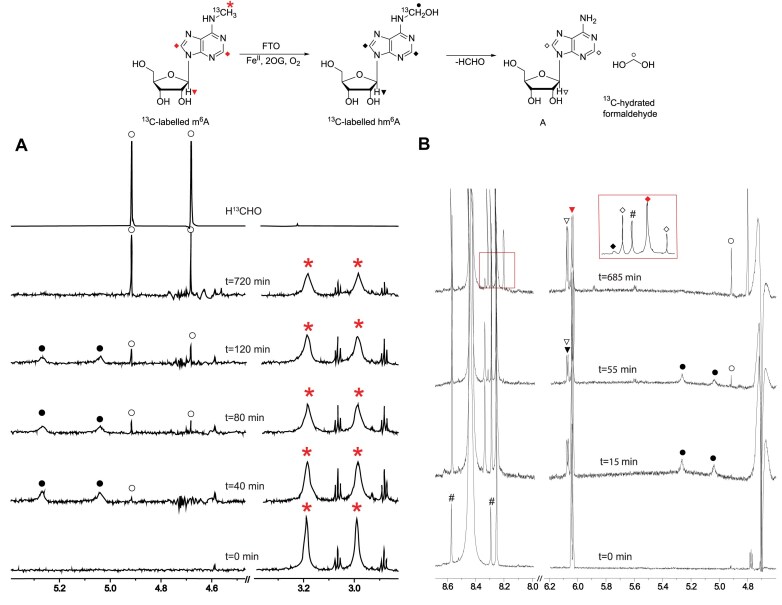
FTO-catalysed ^13^C-m^6^A NMR time course at 310 K. ^1^H resonances bonded to a [^13^C]-carbon appear as doublets due to the ^1^*J*_CH_ coupling. Alternating (**A**) 1D HSQC and (**B**) ^1^H NMR time-course spectra of FTO-catalysed oxidation with ^13^C-m^6^A. Assigned resonances: (i) *δ*_H_ 2.99 ppm and *δ*_H_ 3.19 ppm, ^13^C-labelled methyl m^6^A (red asterisks); (ii) *δ*_H_ 4.68 ppm and *δ*_H_ 4.92 ppm, hydrated ^13^C-formaldehyde (open circles); (iii) *δ*_H_ 5.03 ppm and *δ*_H_ 5.26 ppm, hemiaminal (N-^13^CH_2_OH, solid circles); (iv) *δ*_H_ 6.03 ppm (m^6^A, inverted red triangle) and *δ*_H_ 6.07 ppm (hm^6^A, inverted black solid triangle and A, inverted black triangle), anomeric protons; and (v) *δ*_H_ 8.25 ppm (m^6^A, red rhombus), *δ*_H_ 8.33 ppm (hm^6^A, black solid rhombus), and *δ*_H_ 8.20 ppm and *δ*_H_ 8.31 ppm (A, open rhombus), aromatic protons. Satellite resonances of buffer: #.

We then investigated FTO and ALKBH5 catalysis using selectively ^13^C-methyl-labelled m^6^A (^13^C-m^6^A); gradient-selected 1D heteronuclear single quantum correlation (^1^H–^13^C-HSQC) with ^1^*J*_CH_ optimized to 145 Hz and ^1^H NMR spectra were acquired (at 37°C). With FTO, 15 min after initial mixing (when the first acquisition was complete), the *N*^6^-^13^CH_2_OH group was observed at *δ*_H_ 5.04 ppm and *δ*_H_ 5.26 ppm (^1^*J*_CH_ = 160 Hz). New resonances corresponding to anomeric (*δ*_H_ 6.07 ppm) and aromatic protons of ^13^C-hm^6^A (*δ*_H_ 8.33 ppm) were observed, but resonances corresponding to ^13^C-labelled formaldehyde (^13^CH_2_O) were not observed (Fig. 4), suggesting that the observed product of FTO is hm^6^A in the absence of fragmentation of the hemiaminal. The appearance of the *N*^6^-^13^CH_2_OH resonances correlated with succinate formation and inversely with reduction of the *N*^6^-[^13^C]-CH_3_ resonances. Approximately 40 min after initial mixing, a peak at *δ*_H_ 4.92 ppm was observed, indicating the formation of H^13^CHO; the increase in the ^13^CH_2_OH resonance correlated inversely with a decrease in the *N*^6^ H^13^CHO resonances (Fig. 4). The presence of H^13^CHO was confirmed by spiking with authentic H^13^CHO (Fig. 4). The formation of succinate was observed to halt ∼40 min after the reaction had been started (possibly due to the limited availability of oxygen); however, the H^13^CHO resonances continued to increase even after 600 min after initial mixing (Fig. 4). This observation indicates that the formation of H^13^CHO is, at least partially, a non-enzyme-catalysed process, likely involving gradual hm^6^A fragmentation. We measured the Michaelis–Menten constant, *K*_M_, of m^6^A nucleoside as a substrate of FTO by HPLC and found that it was 791 μM (Supplementary Fig. S8), a relatively high value, suggesting that m^6^A in the nucleoside form is likely not a physiologically relevant substrate in cells. Despite use of various conditions (e.g. varied temperature, ionic strength), ALKBH5 was not observed to catalyse effective oxidation of the ^13^C-m^6^A nucleoside.

**Figure 5.**
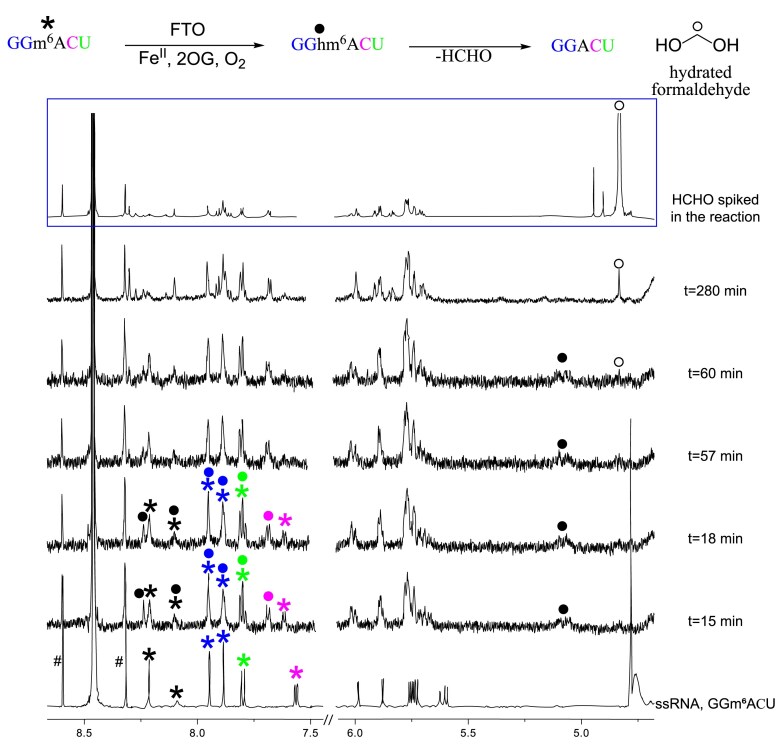
FTO-catalysed ssRNA GGm^6^ACU NMR time course. ^1^H NMR (310 K) time course showing FTO-catalysed hemiaminal (m^6^A) formation. Conditions: FTO (20 μM), 2OG (1 mM), sodium-l-ascorbate (1 mM), Fe(II) ammonium sulfate (80 μM), and GGm^6^ACU (200 μM) in ammonium formate buffer in D_2_O (pD 7.9). The first acquisition (32 scans) was completed 15 min after mixing. The evidence for hemiaminal methylene peak (hm^6^A; black solid circle) was observed as a broad peak at *δ*_H_ 5.1 ppm; hm^6^A slowly fragments into adenosine and formaldehyde (*δ*_H_ 4.88 ppm; black open circle). *In situ* generated formaldehyde resonances matched with the authentic HCHO spiked into the reaction. Aromatic resonances corresponding to nucleosides in GGm^6^ACU (asterisk) appear at (i) *δ*_H_ 7.56 ppm (C, pink), (ii) *δ*_H_ 7.80 ppm (U, green), (iii) *δ*_H_ 7.88 ppm and *δ*_H_ 7.94 ppm (G, blue), and (iv) *δ*_H_ 8.09 ppm and *δ*_H_ 8.21 ppm (m^6^A, black); and GGhm^6^ACU (solid circle) appear at (i) *δ*_H_ 7.68 ppm (C, pink), (ii) *δ*_H_ 7.80 ppm (U, green), (iii) *δ*_H_ 7.88 ppm and *δ*_H_ 7.94 ppm (G, blue), and (iv) *δ*_H_ 8.24 ppm (hm^6^A, black). Satellite resonances of ammonium formate buffer: #. Control experiments’, first (ssRNA) and last (HCHO spiked), spectra consisted of 160 scans.

We next investigated FTO and ALKBH5 catalysis using a 5-mer ssRNA-containing m^6^A (GGm^6^ACU) as a substrate in the presence of 2OG, ascorbate, and ferrous iron in ammonium formate buffer in D_2_O at pD 7.9. With FTO, 15 min after mixing (when the first ^1^H NMR acquisition was complete), a broad resonance at *δ*_H_ 5.1 ppm, corresponding to the hemiaminal methylene of GGhm^6^ACU, was observed. New resonances corresponding to the aromatic protons of GGhm^6^ACU at *δ*_H_ 7.68 ppm (C), *δ*_H_ 7.8 ppm (U), and *δ*_H_ 8.24 ppm (hm^6^A) and overlapping resonances at *δ*_H_ 7.88 ppm (G) and *δ*_H_ 7.94 ppm (G) were observed (Fig. 5). A quantifiable formaldehyde resonance at *δ*_H_ 4.88 ppm was not observed until 1 h after mixing.

For ALKBH5, the initial acquisition was done at 298 K (25 min) to slow catalysis and potentially enable hm^6^A detection; however, with the exception of succinate, new resonances were not observed (Supplementary Fig. S9). The temperature was then increased to 310 K (6 min), and a peak at *δ*_H_ 4.83 ppm corresponding to hydrated formaldehyde was observed after completion of the first acquisition (Supplementary Fig. S9), along with resonances corresponding to anomeric and aromatic protons of (demethylated) GGACU. Peaks corresponding to the hemiaminal methylene of GGhm^6^ACU were not observed throughout the ALKBH5-catalysed reactions, supporting ALKBH5-catalysed demethylation of m^6^A in ssRNA.

We also investigated FTO and ALKBH5 catalysis by NMR at 25°C using an 8-mer ssRNA-containing m^6^A as substrate. We observed production of hm^6^A by FTO, while there was no evidence of hm^6^A production by ALKBH5 (Supplementary Figs S10 and S11), consistent with our results using 5-mer ssRNA.

We investigated whether ALKBH5_66–292_ can catalyse fragmentation of hm^6^A in an 8-mer ssRNA, prepared using FTO, using the IP-RP-LC/ESI-MS method (Supplementary Fig. S12A). After production of hm^6^A, FTO was removed and ALKBH5 was added to the solution. We observed levels of the demethylated product (1261.1766, −2) increased (due to ALKBH5 catalysis), while the hm^6^A (*m*/*z* 1276.1832, −2) levels remained relatively constant. After 15 min, a very low level peak corresponding to a new product, potentially f^6^A (*m*/*z* 1274.9894, −2) was observed (Supplementary Fig. S12B). These observations imply ALKBH5 does not efficiently catalyse fragmentation of hm^6^A in solution, at least under the tested conditions. Note this conclusion does not argue against a protein-bound hm^6^A intermediate during ALKBH5 catalysis, since 2OG oxygenase catalysis (normally) proceeds via a multi-step ordered sequential mechanism [34, 35].

To confirm that ALKBH5 possesses hm^6^A fragmentation activity, we performed QTOF-MS time-course assays on the catalysis of 8-mer m^6^A ssRNA oxidation by ALKBH5_74–292_ wild-type construct and the ALKBH5_74–292_ K132E mutant, in which a critical lysine involved in hm^6^A fragmentation had been mutated, in the presence and absence of inhibitor pydridine-2,4-dicarboxylate (2,4-PDCA), an ALKBH5 inhibitor [33, 55, 56] (Supplementary Figs S13–S16). For the ALKBH5_74–292_ wild-type construct, regardless of the presence of 2,4-PDCA, an increase in demethylated product (*m*/*z* 503.86, −5) but not hm^6^A ssRNA (*m*/*z* 509.86, −5) was observed (Supplementary Figs S13 and S14). While 2,4-PDCA appeared to reduce the activity of wild-type ALKBH5_74–292_, it did not result in accumulation of hm^6^A (Supplementary Fig. S14), suggesting that the absence of hm^6^A is not the result of the high activity of recombinant ALKBH5_74–292_. On the other hand, the ALKBH5_74–292_ K132E mutant, both in the presence and in the absence of 2,4-PDCA, produced hm^6^A (*m*/*z* 509.86, −5) (Supplementary Figs S15 and S16). These results suggest that ALKBH5 is a *bona fide* demethylase that is dependent on Lys132 for its hm^6^A fragmentation activity.

The combined MS and NMR results imply that the catalytic activity of FTO results in m^6^A hydroxylation giving hm^6^A. Although we cannot rule out the possibility that some demethylated product is directly produced at the FTO active site, our results imply most, if not all, of the observed demethylated product observed during FTO catalysis is produced with an observable time delay post-hydroxylation by FTO, likely as a result of non-enzymatic fragmentation of hm^6^A. By contrast with FTO, ALKBH5 is an efficient m^6^A demethylase. Note that although hm^6^A is relatively stable from a kinetic perspective, it is not thermodynamically stable and an excess of HCHO is required to observe substantial formation of hm^6^A under equilibrating conditions, as observed in reported NMR studies [49].

### FTO catalyses oxidation of a 5′-cap mRNA substrate producing hm^6^A_m_

**Figure 6.**
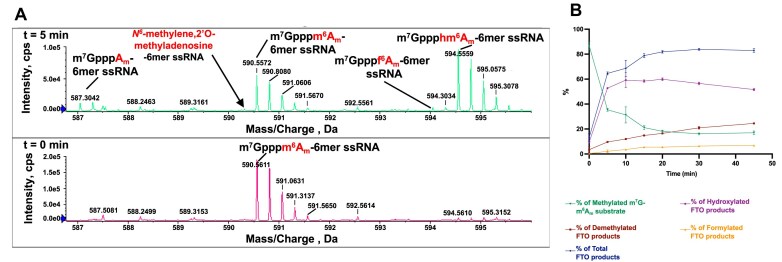
FTO-catalysed oxidation of a 5′-cap substrate. (**A**) QTOF-MS time-course analysis of 6-mer m^7^Gpppm^6^A_m_ ssRNA (m^7^Gpppm^6^A_m_UACUU) oxidation as catalyed by FTOΔ31. The extracted spectra recorded at different time points indicate 6-mer m^7^Gpppm^6^A_m_ ssRNA oligo (*m*/*z* 590.56, −4) is converted to a hm^6^A_m_ product (*m*/*z* 594.56, −4), which was the major observed product throughout the time course (45 min). Peaks for the demethylated product (*m*/*z* 587.06, −4) intensify as the reaction progresses. Evidence for a second apparent oxidation product, f^6^A_m_ (*m*/*z* 594.06, −4), was also observed, albeit at low levels. Similarly to reaction of FTO with the 8-mer m^6^A ssRNA, evidence for an imine species (*m*/*z* 590.31, −4) was not detected (as indicated by arrows). (**B**) Plots showing the reaction product profile for FTOΔ31-catalysed oxidation of 6-mer m^7^Gpppm^6^A_m_ ssRNA, (generated using GraphPad Prism 10.1.1). Reactions were performed in triplicate (*n* = 3); error bars represent the standard error of the mean (SEM).

There is evidence that the reaction outcomes of some 2OG oxygenases are affected by context, e.g. substrate length or conformation with polymeric substrates [31, 63]. We were thus interested to study the FTO reaction products, in particular with respect to whether hydroxylation or demethylation occurs, with m^6^A_m_, a reported FTO substrate adjacent to the 5′ *N^7^*-methylguanosine (m^7^G) cap [27, 28]. To investigate this, we tested 6-mer m^7^Gpppm^6^A_m_ ssRNA (m^7^Gpppm^6^A_m_UACUU) as a substrate of FTOΔ31 and ALKBH5_74–292_, with incubation conditions as before analysing by SCIEX Triple TOF 6600 (QTOF) mass spectrometer. As reported [27, 28], ALKBH5_74–292_ was not observed to catalyse oxidation of m^7^Gpppm^6^A_m_ ssRNA (Supplementary Fig. S17). Consistent with the studies of full-length FTO and FTOΔ31 with the 8-mer m^6^A ssRNA substrate, with FTOΔ31 and m^7^Gpppm^6^A_m_UACUU, the hm^6^A_m_ product (*m*/*z* 594.56, −4) dominated at all time points (Fig. 6A and B, and Supplementary S18). By contrast with hm^6^A_m_, levels for which decreased after 20 min, masses corresponding to the demethylated (A_m_) (*m*/*z* 587.06, −4) and (apparent) formylated (f^6^A_m_) products (*m*/*z* 594.06, −4) increased over time (Fig. 6B), though internal f^6^A was only observed at low levels (Fig. 2A). These observations suggest that FTO could produce a relatively stable hm^6^A_m_ by the 5′ cap of RNA, the potential biological roles of which require further investigation.

### Non-enzyme-catalysed fragmentation of FTO products

**Figure 7.**
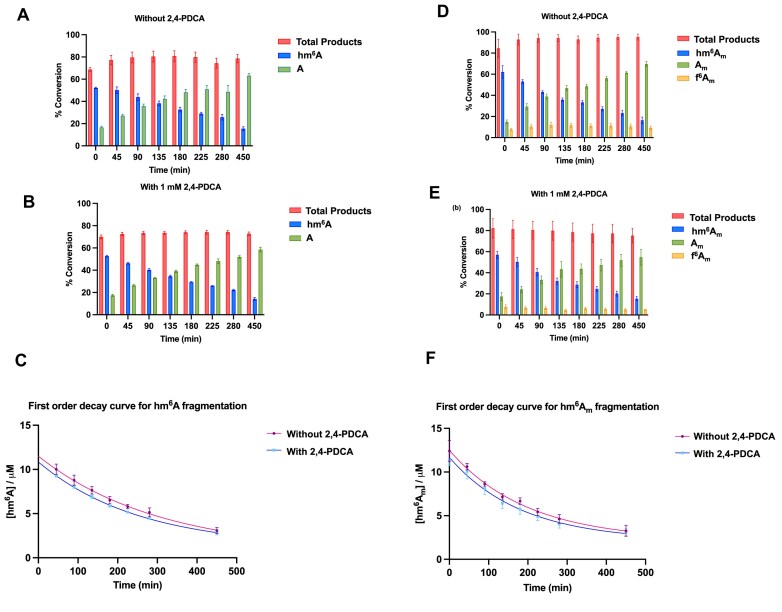
Non-enzymatic fragmentation of hm^6^A/hm^6^A_m_ produced by FTO catalysis. FTOΔ31-catalysed oxidation of 8-mer m^6^A ssRNA oligo (UGGm^6^ACUGC) for 30 min results in ∼50% conversion to hm^6^A. FTO products were monitored over 450 min by QTOF-MS: (**A**) without 2,4-PDCA and (**B**) with 1 mM 2,4-PDCA. Bar graphs (generated using GraphPad Prism 10.1.1) show the presence of 2,4-PDCA does not substantially affect the rate of hm^6^A fragmentation to *N*^6^-demethylated 8-mer ssRNA, indicating that hm^6^A fragmentation is, at least mostly, not enzyme catalysed. Error bars represent SEM of duplicates (*n* = 3). (**C**) [hm^6^A] versus time plot for first-order kinetics of hm^6^A degradation, with and without 2,4-PDCA (generated using GraphPad Prism 10.1.1). Data were fitted into a one-phase decay model. Error bars represent SEM (*n* = 3). Both reactions show similar trends for non-enzymatic fragmentation of hm^6^A and follow first-order kinetics with similar decay rate constants. After initial hydroxylation of 6-mer m^7^Gpppm^6^A_m_ ssRNA (m^7^Gpppm^6^A_m_UACUU) as catalysed by FTOΔ31 (∼50% hm^6^A_m_ generated after 10 min), the sample was divided into two portions and treated with the FTO inhibitor 2,4-PDCA (**E**) or a buffer control (**D**). Products were monitored by QTOF-MS over 450 min. Both reactions show similar profiles, implying that hm^6^A_m_ fragmentation is not enzyme mediated. (**F**) [hm^6^A_m_] versus time plots for first-order kinetics of hm^6^A_m_ fragmentation with and without the addition of 2,4-PDCA. Data were fitted with a one-phase decay model; curves were generated using GraphPad Prism 10.1.1. Reactions were performed in triplicate (*n* = 3); error bars represent the SEM. The fragmentation of hm^6^A_m_ adheres to first-order decay kinetics and exhibits comparable decay profiles with or without 2,4-PDCA, implying that FTO does not catalyse hm^6^A fragmentation.

We then carried out experiments to investigate whether the relatively low levels of *N*^6^-demethylated 8-mer ssRNA and demethylated m^7^Gpppm^6^A_m_UACUU produced in FTO reactions are due to enzyme- or non-enzyme-mediated reactions. Thus, FTOΔ31 was incubated with 8-mer internal m^6^A ssRNA and m^7^Gpppm^6^A_m_UACUU separately for 30 min as previously described. 2,4-PDCA, a known FTO inhibitor [55], or a control solution was then added and the reactions were analysed by QTOF-MS as before. In both the 2,4-PDCA and control treated samples, the hm^6^A levels were similar at the same time points (Fig. 7A and B). The demethylated product levels manifested an increase over time, consistent with studies on hm^6^A stability [49] and its non-enzyme-catalysed fragmentation with or without FTO. We examined the kinetics of hm^6^A fragmentation at pH 7.2 (pH in the nucleus), observing first-order kinetics with similar decay constants in the presence (3.9 × 10^−3^ min^−1^) and absence (3.2 × 10^−3^ min^−1^) of 2,4-PDCA. Under these conditions, the half-lives of hm^6^A with and without 2,4-PDCA were calculated as 176 and 211 min, respectively (Fig. 7C).

We studied the fragmentation of FTOΔ31 generated 5′ m^7^G triphosphate hm^6^A_m_ cap into A_m_ by adding 2,4-PDCA or a control solution after 10 min incubation, a time when hm^6^A_m_ product levels were maximal (Fig. 7D and E). Both the 2,4-PDCA-treated and non-PDCA-treated samples manifested similar first-order decay rates for the hm^6^A_m_ product, with decay constants/half-lives of 5.4 × 10^−3^ min^−1^/128 min and 5.0 × 10^−3^ min^−1^/138 min, respectively, under these conditions (Fig. 7F). As before, these observations imply that hm^6^A_m_ fragmentation is, at least predominantly, not FTO-catalysed, supporting the proposal that FTO acts as a hydroxylase within the context of m^6^A_m_ oxidation adjacent to the 5′ cap.

### AlkB, ALKBH2, and ALKBH3 do not act as m^1^A hydroxylases

To investigate whether the hydroxylase activity of FTO is unusual, we carried out studies with DNA damage repair 2OG oxygenases (Fig. 1D), i.e. human ALKBH2 and ALKBH3 and their bacterial homologue AlkB using 1mA ssDNAs as substrates, with analysis by ion-paired ESI-MS (Supplementary Figs S19–S21). Note that some reported ALKBH2/3 and AlkB studies have used substrate digestion and monitoring through LC or MS [40, 43], a method that can result in the loss of hemiaminal detection. The products of undigested ALKBH2/3 and AlkBΔN11 reactions were analysed by IP-RP-LC/ESI-MS. We observed evidence for demethylated adenosine without evidence for *N*^1^-hydroxymethyldeoxyadenosine (1hmA), *N^1^*-formyldeoxyadenosine, or imine products in all three cases (Supplementary Figs S19–S21). Thus, ALKBH2/3 and AlkB converted an m^1^A-containing ssDNA substrate to the demethylated products; note these observations are consistent with knowledge that 1hmA is relatively less stable compared to 6hmA [49].

## Discussion

The MS and NMR assay results presented here support previous studies showing both FTO and ALKBH5 act on internally located m^6^A in ssRNA [14, 15]. In accord with prior studies, FTO, but not ALKBH5, catalyses oxidation of m^7^Gpppm^6^A_m_ ssRNA [27, 28] (Fig. 6 and Supplementary Fig. S18). However, whereas with ALKBH5 the only observed product with m^6^A (8-mer m^6^A ssRNA) was the demethylated adenosine (Supplementary Figs S1B and S13), with FTO both substrate types produced a hemiaminal (hm^6^A/hm^6^A_m_) species as the dominant product at all time points (Figs 2–6 and Supplementary Figs S1, S2, S4, and S18). Both the NMR and MS evidence showed that at early time points, FTO produces a hemiaminal species as the only observed product, without the presence of a demethylated product. Support for the unusual nature of FTO catalysis comes from studies with human ALKBH2 and ALKBH3 and bacterial AlkB, which manifested demethylation reactions with m^1^A-containing substrates (Supplementary Figs S19–S21). Our results also showed that while FTO can act on m^6^A nucleoside, ALKBH5 did not show activity against m^6^A nucleoside. Kinetic studies of FTO on m^6^A nucleoside showed that its *K*_M_ is 791 μM, a level much higher than expected for m^6^A nucleosides in cells [64, 65], suggesting that FTO likely does not function in oxidizing *N*^6^-methyl of m^6^A nucleosides in cells.

The results of studies where FTO reactions with internal m^6^A or m^7^Gpppm^6^A_m_ substrates were halted by the addition of an inhibitor [55] imply that the slow fragmentation of FTO-produced hm^6^A/hm^6^A_m_ giving the demethylated product and formaldehyde that is observed in the presence of FTO is not enzyme catalysed (Fig. 7A–F). ALKBH5 did not catalyse fragmentation of hm^6^A produced by FTO catalysis in solution (Supplementary Fig. S12). This could be because ALKBH5 prefers binding to m^6^A-containing ssRNA more so than the hm^6^A-containing ssRNA. Note, however, that the normally ordered sequential nature of 2OG oxygenase catalysis means protein-bound hm^6^A may be a protein-bound intermediate during ALKBH5 catalysis. Our observations are consistent with prior studies demonstrating the enhanced stability of exocyclic N-linked hemiaminals compared to endocyclic nitrogens, though the latter are faster to form in reactions with formaldehyde [49, 51]. Catalysis by a mutant ALKBH5 K132E demonstrated that the N-linked hemiaminal can be detected by MS, indicating that wild-type ALKBH5 is a *bona fide* m^6^A demethylase.

The combined results strongly indicate that FTO acts as a hydroxylase, initially at least, producing hemiaminal-type products. Further work is required to validate reports that FTO-catalysed reactions on m^7^Gpppm^6^A_m_-containing ssRNA can produce low levels of f^6^A_m_ [32, 33, 48, 49], likely produced by hm^6^A_m_ oxidation (Fig. 6 and Supplementary Fig. S18). Note that the low levels of f^6^A_m_ produced mean that as yet we have been unable to validate its proposed structure by NMR. Further, clear evidence for the formylated f^6^A product was not apparent with the ssRNA internal m^6^A substrate (Fig. 2 and Supplementary Figs S1 and S4). In some MS analyses, there was also evidence for *N*^6^-methyleneadenosine/imine production [32], though it is uncertain whether this is a result of enzyme catalysis and/or due to dehydration from hm^6^A/hm^6^A_m_ under the MS analytical conditions; we did not see evidence for imine formation by NMR, though this could be because it was not present in sufficiently high levels.

If the formation of f^6^A/f^6^A_m_ can be validated in cells, FTO will be one of the set of 2OG oxygenases catalysing sequential oxidations on the same methyl group-derived carbon. Such activities were observed in early studies on 2OG oxygenases involved in gibberellin and cephalosporin biosynthesis [31, 66–68] and have been more recently observed in the ten-eleven translocation enzyme (TET)-mediated oxidation of 5-methylcytosine (5mC) in DNA, where sequential oxidations of a methyl group to an alcohol, then aldehyde, and then acid are observed (Fig. 1E) [69, 70]. Given that we observed evidence for low levels of f^6^A, it is of interest that studies on the TET enzymes report that conversion of 5hmC to 5fC is slow compared to the initial oxidation of 5mC to 5hmC [32].

Most biological studies on FTO and ALKBH5 have focused on their roles as demethylases, affecting function (e.g. to modulate mRNA stability) by removal of m^6^A methyl groups [15, 27, 30, 71–73]. Such roles are difficult to dissect, because they likely also involve m^6^A methyltransferases and m^6^A binding proteins [74]. One possibility is that the reactive products, including formaldehyde and hm^6^A, produced by FTO-catalysed oxidations have functional roles, e.g. to enable cross-linking reactions with nucleic acids, proteins, or small molecules, as well precedented in hemiaminal/Schiff base (bio)chemistry [32, 49]. Although the FTO-catalysed production of hm^6^A/hm^6^A_m_ is striking, in a localized context, HCHO produced by ALKBH5 also has potential to enable similar reactions. Recent work has also shown that f^6^A has potential to transfer a formyl group or undergo hydrolysis to produce formate [75]. Another possibility is that the transient reversible reaction of nucleophilic sites on proteins/nucleic acids with aldehydes serves to hinder irreversible/less reversible reactions, e.g. regulatory N-methylation or damaging alkylation [76]. It should also be noted that there is evidence that, at least in some cases, the substrate and product selectivities of 2OG oxygenases can vary with context, meaning the observations with isolated enzymes may not reflect *in vivo* activities [77], where other factors may alter substrate and product selectivities.

The combined results demonstrate a clear difference in the product selectivities of FTO and ALKBH5, at least in the case of studies with the isolated enzymes. How our biochemical observations relate to the proposed roles for FTO and ALKBH5 in healthy biology and disease is presently unclear. Among multiple reports, both FTO and ALKBH5 are linked to cancer, FTO is linked to obesity [16, 23], and ALKBH5 is linked to regulation of the hypoxic response and is a hypoxia-inducible factor target gene, i.e. is upregulated in hypoxia [78]. A recent study reports increased levels of cerebral formaldehyde in mice after acute high‐altitude hypoxia exposure [78–80]. Mutations/variations in the *FTO*, but not the *ALKBH5*, gene are linked to diabetes and obesity [16, 23, 81], and it is possible that the different product selectivities of the two oxygenases reflect their different biological roles in disease. Given the role of aldehyde chemistry in diabetes [76], the ability of FTO to form hm^6^A is of particular interest in this regard and is the subject of ongoing research. There are also reports linking FTO to aldehydes and alcohol consumption [82]. Further molecular investigations on the potential roles of ALKBH5, FTO, and other formaldehyde/hemiaminal producing oxygenases in the context of the physiological hypoxic response are thus of interest.

## Supplementary Material

gkaf813_Supplemental_File

## Data Availability

All data relevant to the results are included within the manuscript. MS and NMR data are available upon request.

## Notes

Present address: California Institute for Biomedical Research, La Jolla, CA 92037, United States
