# Supplementary material for: Evidence human FTO catalyses hydroxylation of N6-methyladenosine without direct formation of a demethylated product contrasting with ALKBH5/2/3 and bacterial AlkB
Source: Nucleic Acids Res. 2025 Aug 28;53(16):gkaf813. doi: 10.1093/nar/gkaf813 (PMC12392094; doi:10.1093/nar/gkaf813)
Supplement: gkaf813_Supplemental_File [file gkaf813_supplemental_file.pdf]

Supplementary Information

Evidence human FTO catalyses hydroxylation of N^6-methyl adenosine without direct formation of a demethylated product contrasting with ALKBH5/2/3 and bacterial AlkB

Simranjeet Kaur[1,#], Pratheesh Maheswaran[2,#], Samanpreet Kaur[2], Yingqi Lai[1], Eidarus Salah[2], Dong Zhang[2], Shifali Shishodia[2,3,*], Christopher J. Schofield[2,*], Wei Shen Aik[1,*]

[1]Department of Chemistry, Hong Kong Baptist University, Kowloon Tong, Hong Kong SAR, China.

[2]The Department of Chemistry and the Ineos Oxford Institute for Antimicrobial Research, Chemistry Research Laboratory, University of Oxford, 12 Mansfield Road, Oxford, OX1 3TA, United Kingdom.

[3]Current address: California Institute for Biomedical Research, La Jolla, CA 92037, United States.

[#] [#] The authors wish it to be known that, in their opinion, the first two authors should be regarded as Joint First Authors.

* To whom correspondence should be addressed. Tel: +852 34116682; Fax: +852 34117063; Email: aikweishen@hkbu.edu.hk. Correspondence may also be addressed to Christopher J. Schofield. Tel: +44 01865 275 625; Fax: +44 01865 285 002; Email: christopher.schofield@chem.ox.ac.uk. Correspondence may also be addressed to Shifali Shishodia. Email: sshishodia@scripps.edu

SUPPLEMENTARY DATA

Figures S1 to S21

Supplementary References

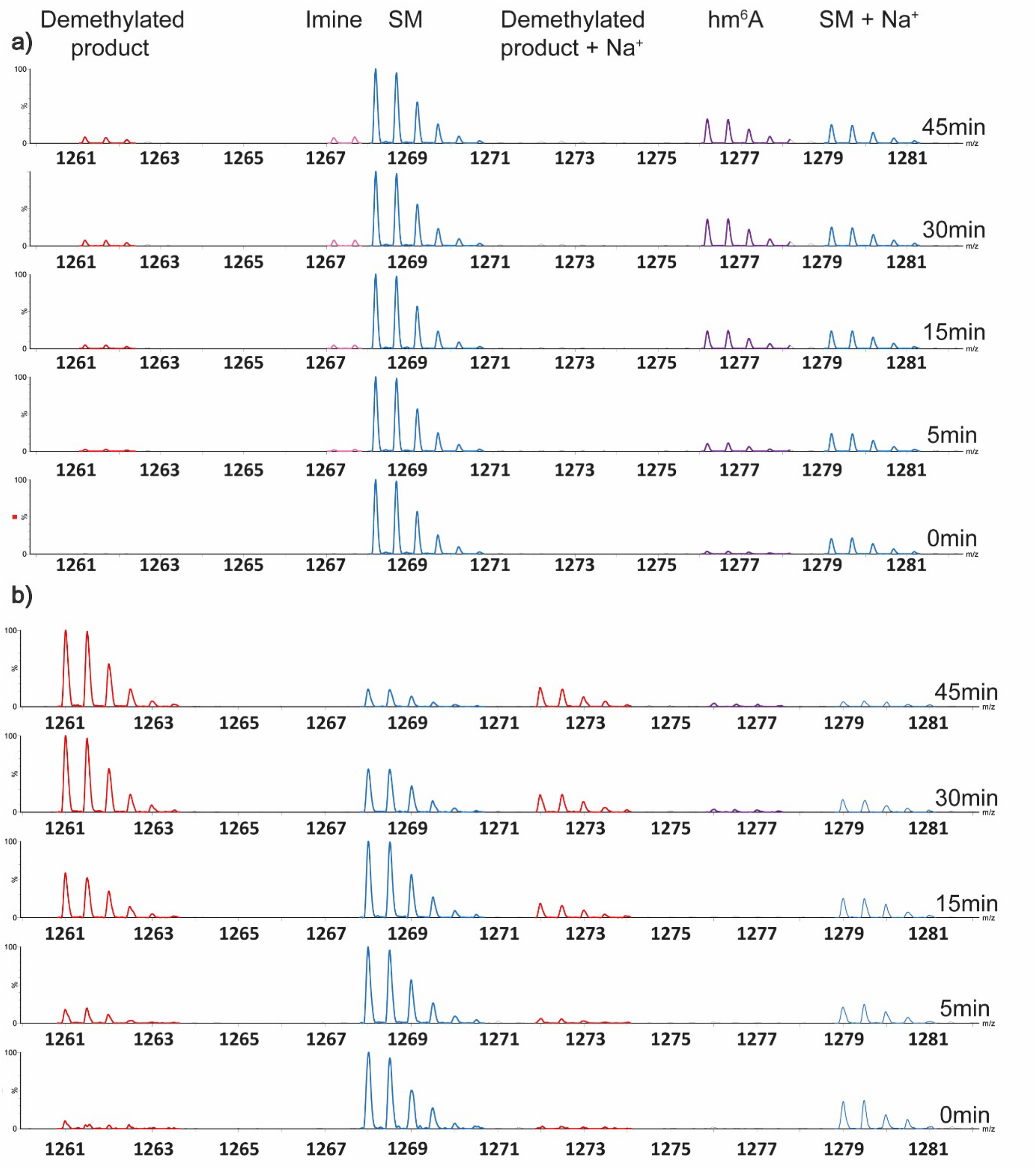

Figure S1. Evidence FTO acts as hydroxylase and ALKBH5 as a demethylase. (a) IP-RP-LC/ESI-MS time course analysis of ssRNA UGGm^{6}ACUGC oxidation as catalysed by full-length FTO. The extracted ion spectra imply UGGm^{6}ACUGC (m/z 1268.1766, -2 charge state, blue) is converted to hm^{6}A (m/z 1276.1832, -2, purple), which was the major observed product throughout the time-course (45 min). Masses corresponding to low levels of the N^6-demethylated (m/z 1261.1766, -2, red) and, potentially, imine (m/z 1267.6827, -2, pink) products were observed; no evidence for f^{6}A formation was accrued under the tested conditions. SM: starting material. (b) IP-RP-LC/ESI-MS time course analysis of ssRNA UGGm^{6}ACUGC oxidation as catalysed by ALKBH5. The extracted ion spectra imply that UGGm^{6}ACUGC (m/z 1268.1766, -2 charge state, blue) is converted to the N^6-demethylated product (m/z 1261.1766, -2, red), which was the major observed product throughout the time-course (45 min). A low level of a mass corresponding to hm^{6}A (m/z 1276.0066, -2) was observed for the 30/45 min ALKBH5 time points, possibly reflecting non-enzymatic reaction of HCHO produced by demethylation. Evidence for formation of f^{6}A was not accrued under the tested conditions. SM: starting material. See Fig. S2, for time-course and repeats.

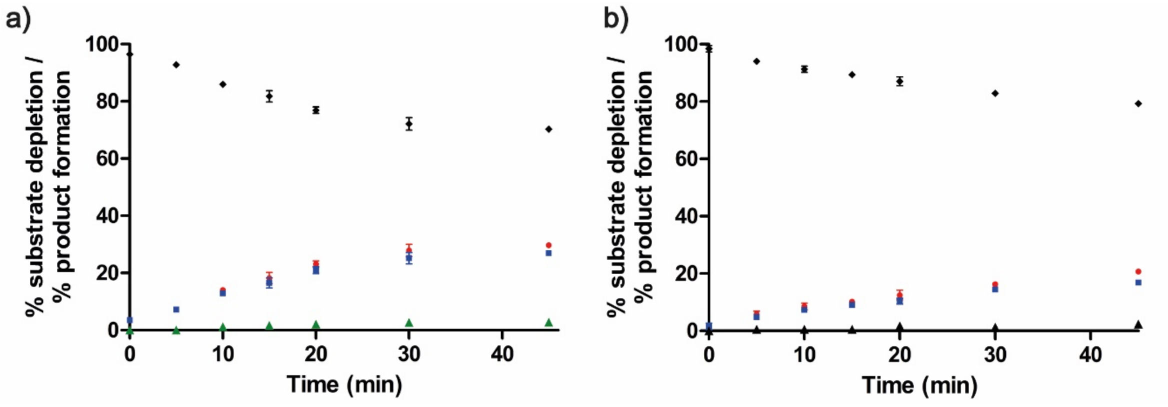

Figure S2. FTO time-course analysis. IP-RP-LC/ESI-MS analysis at 0.5 µM (**a**) and 0.25 µM (**b**) FTO, analysing product formation. Black diamonds represent substrate depletion; red circles indicate total product; blue squares denote hydroxylated product (hm^6A); and green diamonds indicate the demethylated product. Reactions were performed in triplicate (n = 3); error bars represent the standard error of the mean (SEM). Data were analysed using GraphPad Prism 5.04.

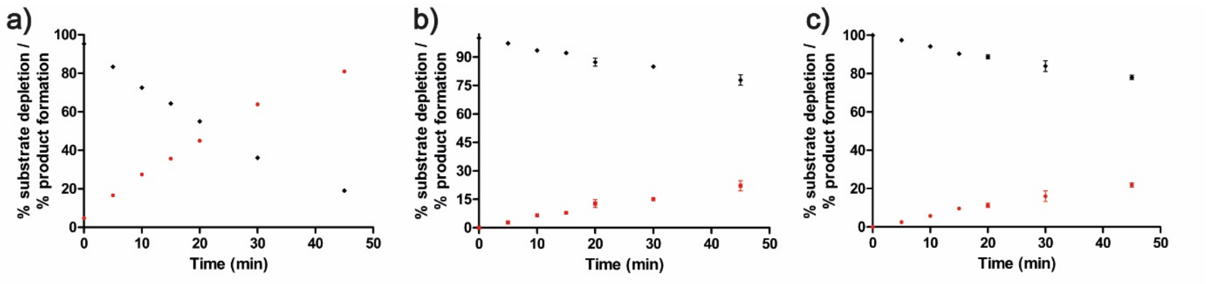

Figure S3. ALKBH5 time-course analysis. IP-RP-LC/ESI-MS analysis at 8 µM (**a**), 4 µM (**b**), and 2 µM (**c**), showing product percentages. Black diamonds represent substrate depletion; red circles indicate the demethylated product. Reactions were performed in triplicate (n = 3); error bars represent the standard error of the mean (SEM). Data were analysed using GraphPad Prism 5.04.

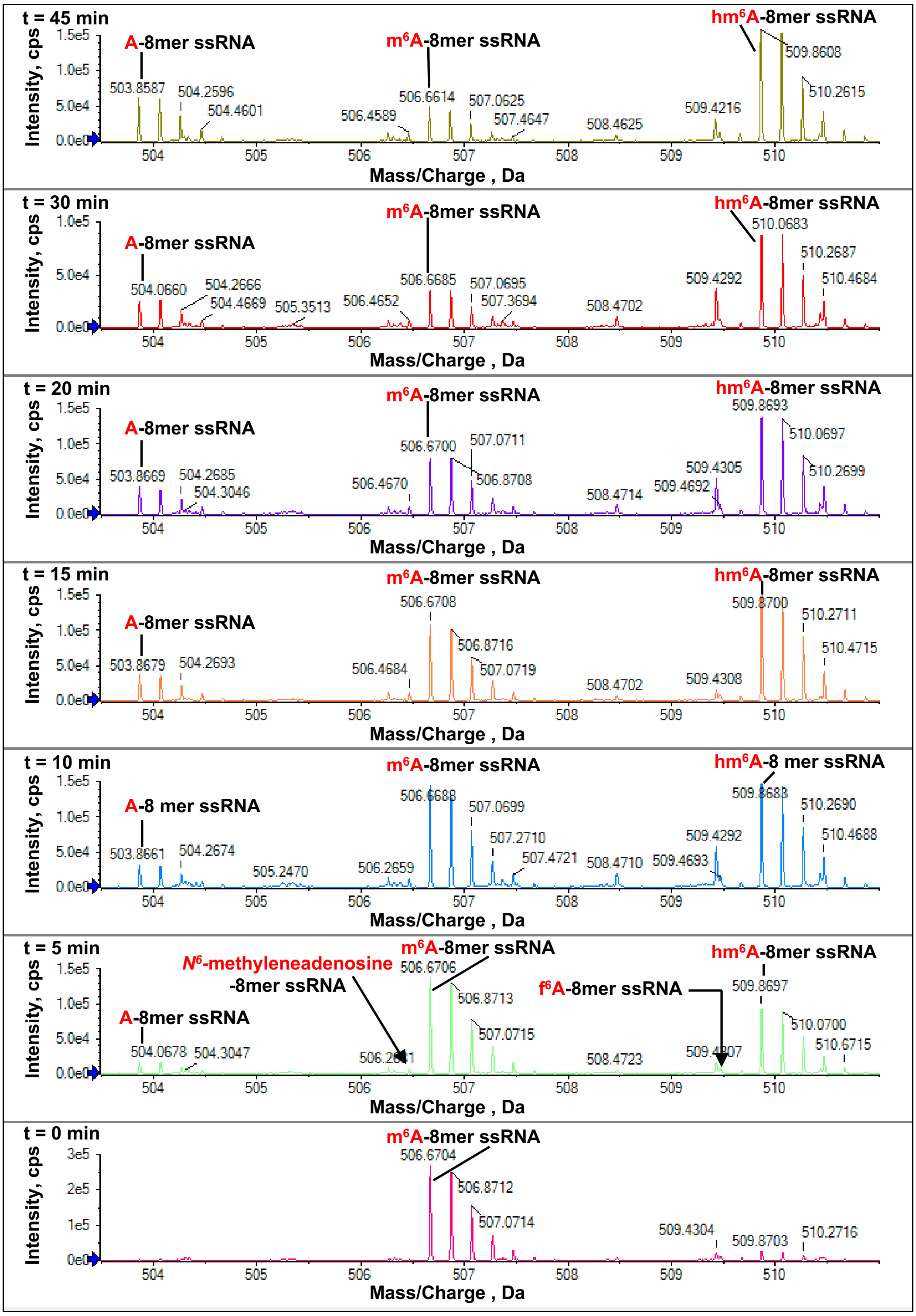

Figure S4. QTOF-MS time course analysis for oxidation of an 8-mer ssRNA oligonucleotide with internal m^{6}A (UGGm^{6}ACUGC) as catalysed by FTOΔ31. The extracted spectra recorded at different time points (0-45 min) indicate that the 8-mer m^{6}A ssRNA oligomer (m/z 506.67, -5) is converted to a hydroxylated product (m/z 509.86, -5), which was observed as the major product throughout the reaction time-course. The peak corresponding to the demethylated product (m/z 503.86, -5) intensifies as the reaction progresses. Substantial peaks corresponding to f^{6}A (m/z 509.46, -5) (as indicated by black arrows) were not observed.

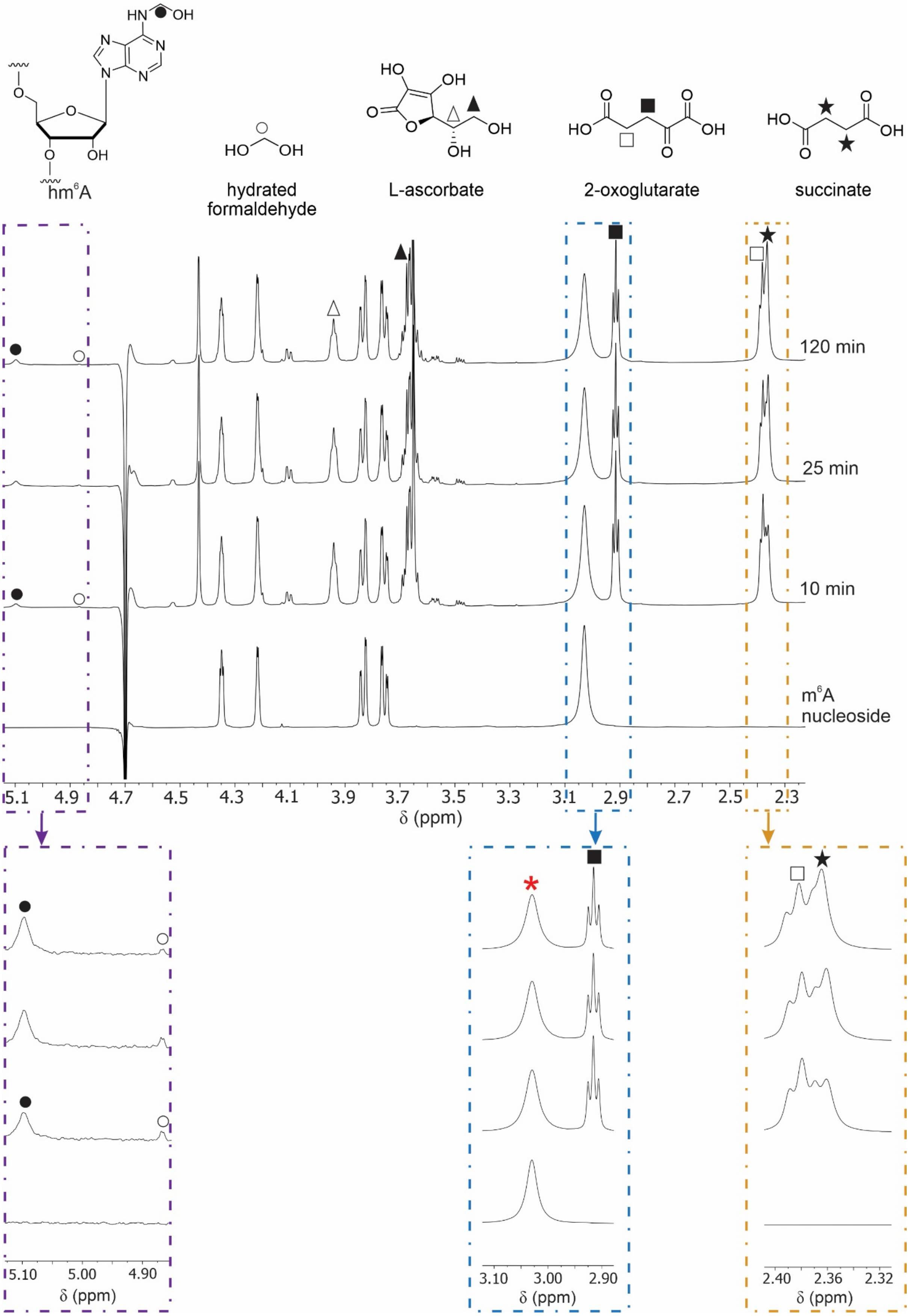
hm6A
hydrated formaldehyde
L-ascorbate
2-oxoglutarate
succinate
120 min
25 min
10 min
m6A nucleoside
δ (ppm)

Figure S5. NMR evidence FTO is a hydroxylase when using the m^{6}A nucleoside as a substrate. ^{1}H NMR time-course spectra (700 MHz) of m^{6}A nucleoside oxidation as catalysed by full-length FTO are shown. The decrease in the broad resonance at δ_H 3.02 ppm (red asterisk), corresponding to the N^6-CH_3 group of the m^{6}A nucleoside indicates FTO mediated oxidation. The peak at δ_H 5.10 ppm (black circle) corresponds to the hm^{6}A hemiaminal and increases in intensity from 10 to 120 min. The low intensity peak at δ_H 4.87 ppm (open circle) indicates the presence of hydrated HCHO. The decrease in the 2OG methylene peaks at δ_H 2.93 ppm (black square) and 2.37 ppm (open square), along with the increase in the succinate peak at δ_H 2.34 ppm (black star), supports FTO-catalysed 2OG oxidation. The results are in accord with those reported (52). The low levels of hydrated formaldehyde observed, are likely in part derived from the ammonium formate buffer used via reduction involving Fe(II) ammonium sulfate.

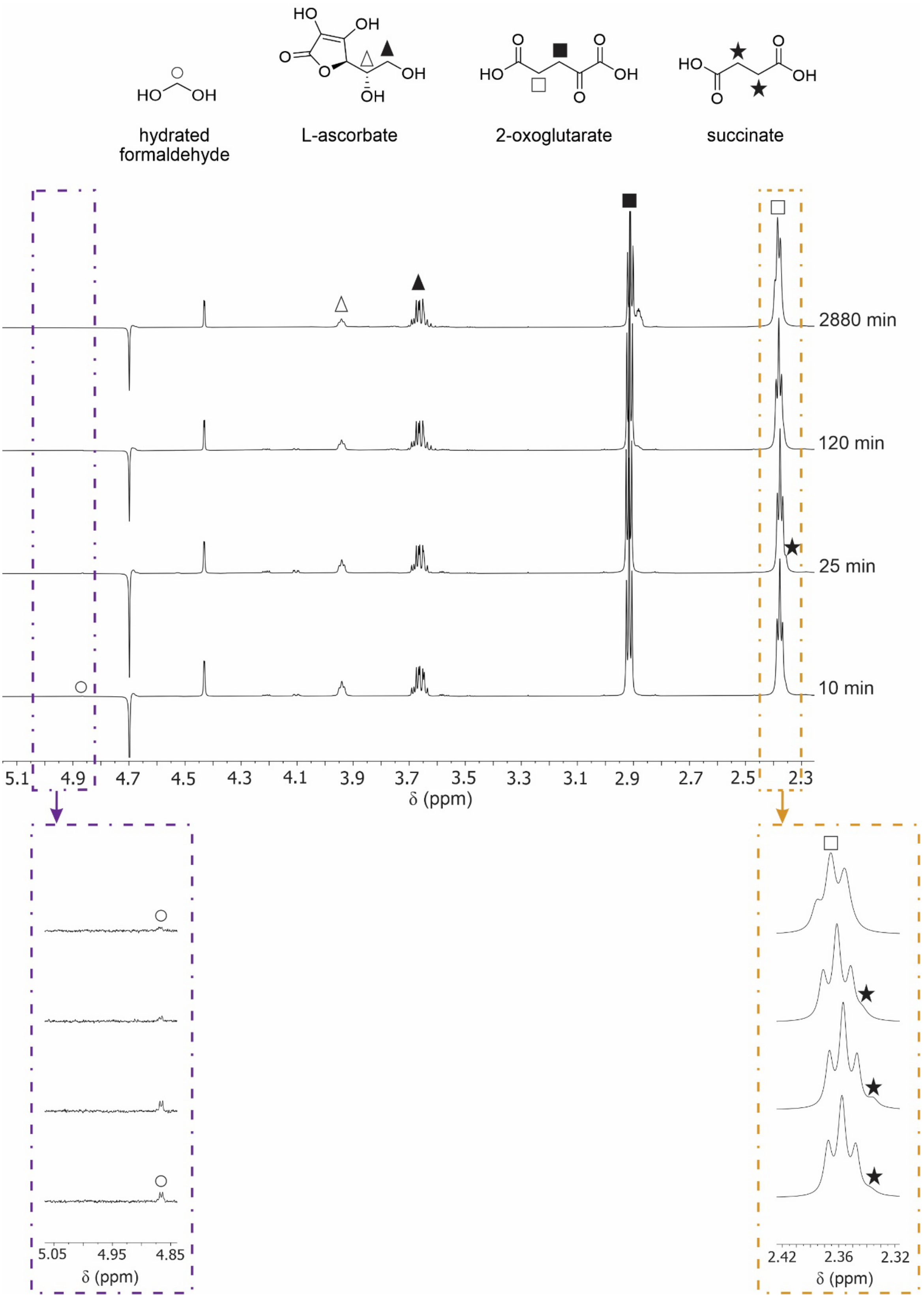

Figure S6. NMR analysis of 2OG turnover in the presence of FTO and the absence of substrate. ^{1}H NMR (700 MHz) time-course spectra of full-length FTO catalysed reaction of 2OG in the absence of substrate. There is a slight decrease in 2OG peaks at δ_H 2.93 ppm (black square) and 2.37 ppm (open square), along with a small increase in the succinate peak at δ_H 2.34 ppm (black star), suggesting low levels of substrate uncoupled turnover of 2OG. Note that the succinate peak at 2880 min likely moves further downfield merging with the 2OG peak. Low levels of hydrated formaldehyde were observed in the absence of substrate, likely derived from the (ammonium formate) buffer used via reduction involving Fe(II) ammonium sulfate.

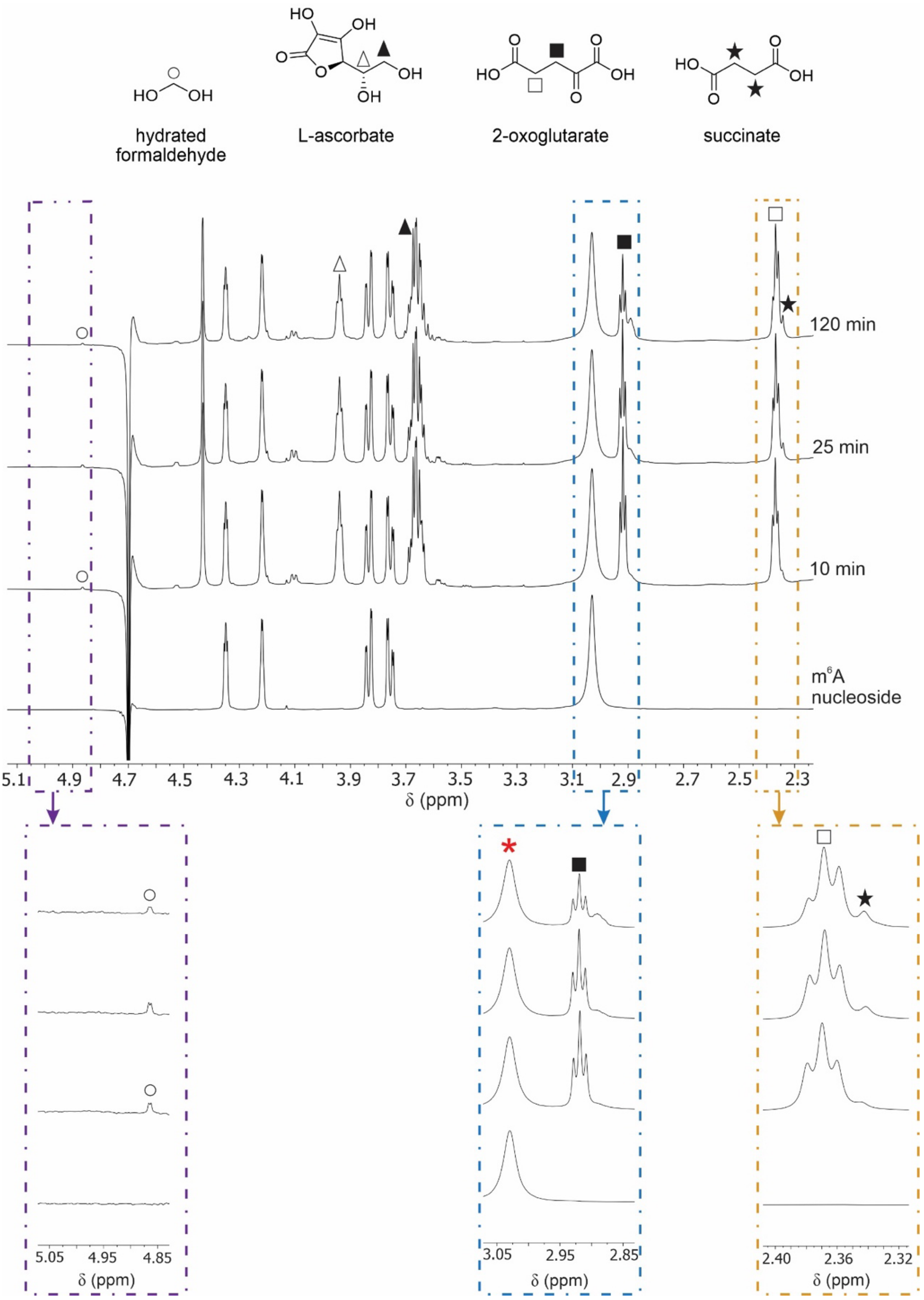

Figure S7: NMR evidence for the inability of ALKBH5 to efficiently catalyse m⁶A nucleoside oxidation. ^{1}H NMR (700 MHz) time-course spectra of potential m⁶A nucleoside oxidation by ALKBH5 are shown. The broad resonance at δ_H 3.02 ppm (red asterisk), corresponding to the *N*^{6}-CH_3 group of the m⁶A nucleoside, remains unchanged over 120 minutes. No evidence for hemiaminal formation was observed. The small decrease in the 2OG peaks at δ_H 2.92 ppm (black square) and 2.38 ppm (open square), along with a small increase in the succinate peak at δ_H 2.34 ppm (black star), indicates possible ALKBH5, or non-enzymatic, substrate-uncoupled turnover of 2OG. Low levels of hydrated formaldehyde were observed, likely derived from the (ammonium formate) buffer used via reduction involving Fe(II) ammonium sulfate.

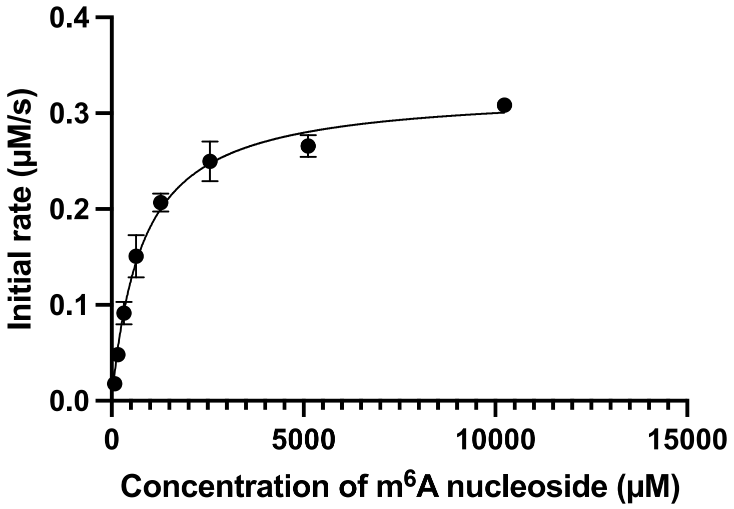

Fig. S8. Michaelis-Menten analysis of m⁶A nucleoside as a substrate for FTO. Michaelis-Menten constant, K_M = 791 µM; maximal velocity, V_{max} = 0.324 µM/s.

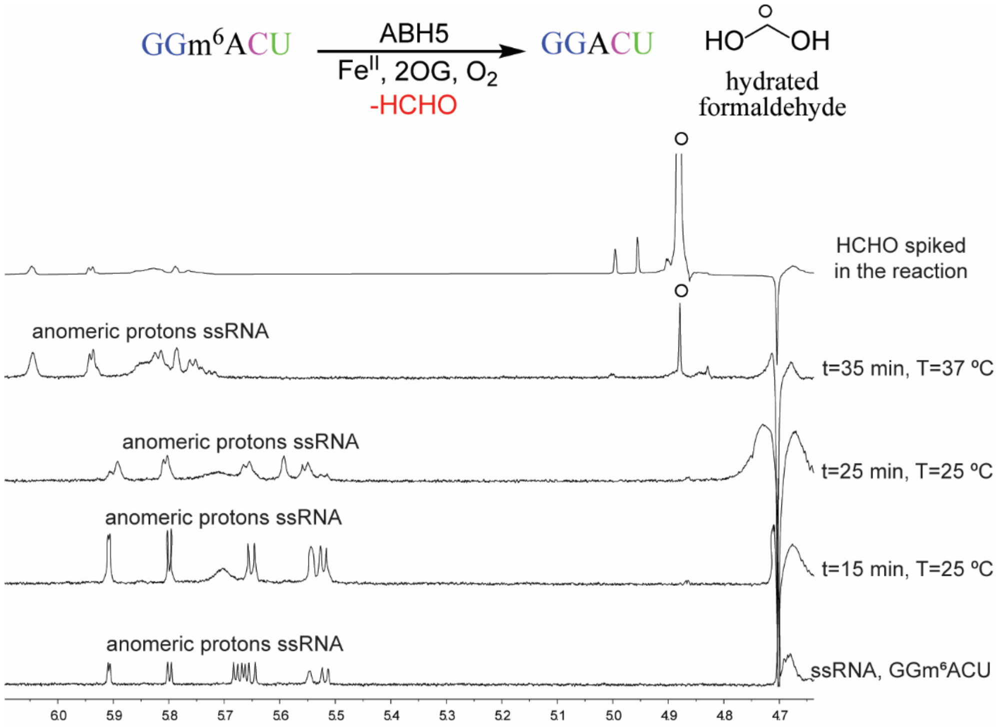

Figure S9. ALKBH5-catalysed ssRNA GGm^{6}ACU NMR time course. ^{1}H NMR time course showing ALKBH5 catalysed formaldehyde (circle) formation. A solution of ALKBH5 (20 µM), 2OG (1 mM), L-ascorbate (1 mM), Fe(II) ammonium sulfate (80 µM), GGm^{6}ACU (200 µM) was prepared in ammonium formate buffer in D_2O, pD 7.9; the first acquisition (16 scans) was completed 15 min after mixing. No resonances corresponding to a hemiaminal were observed, even at the lower temp. (298 K). The temperature of the probe was raised to 310K (6 min). The formaldehyde resonance at δ_H 4.83 ppm were apparent after the first acquisition was completed at 310K. In situ generated formaldehyde resonances matched with the authentic HCHO spiked into the reaction.

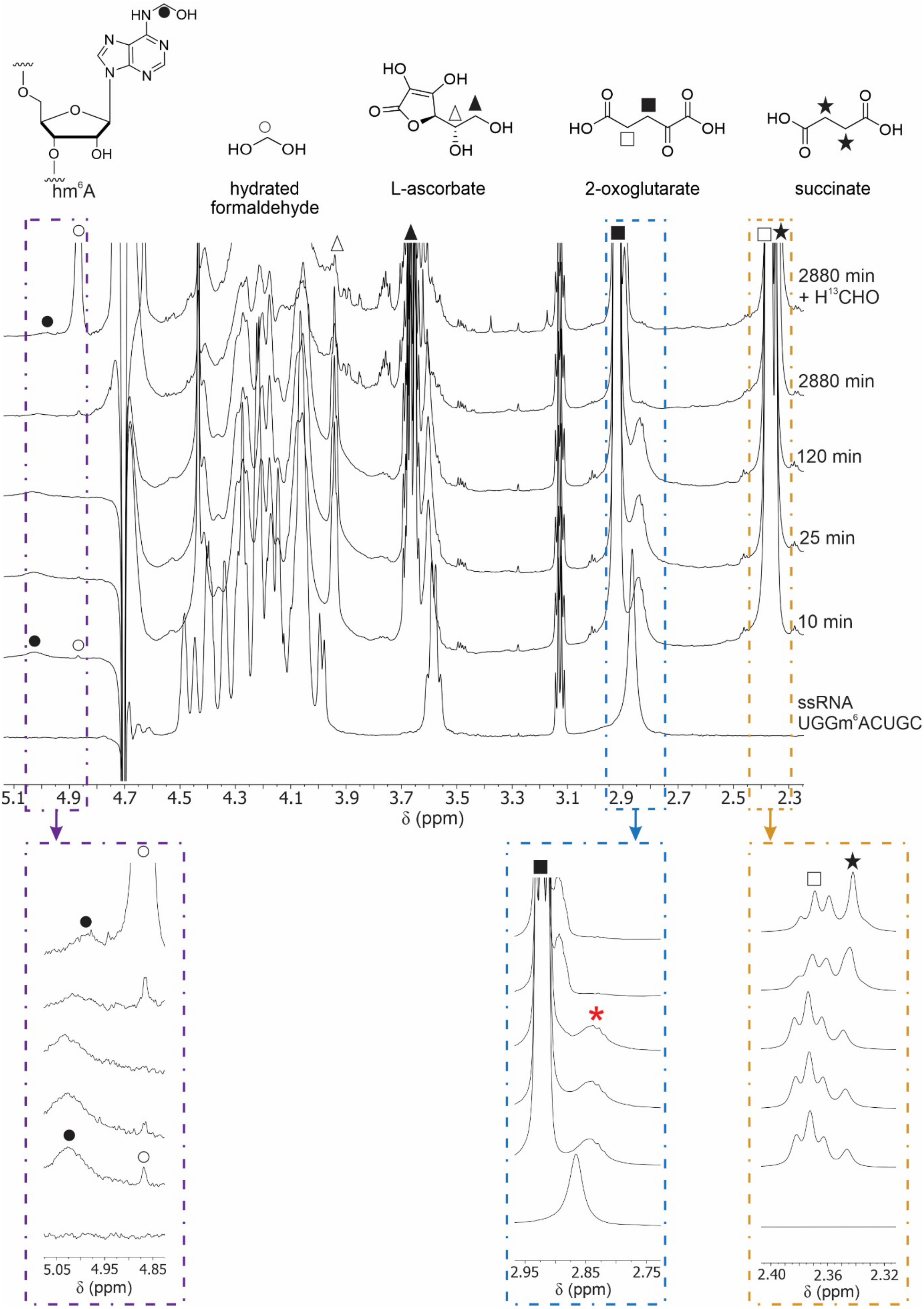

Figure S10. NMR evidence that FTO is a hydroxylase using ssRNA UGGm⁶ACUGC as a substrate. ^{1}H NMR (700 MHz) time-course spectra of ssRNA UGGm⁶ACUGC oxidation catalysed by full-length FTO are shown. The decrease in the N^{6}-CH_3 resonance at δ_H 2.85 ppm (red asterisk), corresponding to the methyl group of ssRNA UGGm⁶ACUGC, over 120 min with complete consumption by 2880 min indicates FTO-mediated oxidation. The

broad peak at δ_H 5.02 ppm (black circle), observed after 10 min, suggests formation of a hemiaminal; the peak intensity increases over 120 min. An additional time point at 2880 min indicates hemiaminal stability. Low levels of hydrated formaldehyde were likely derived (at least in part) from the (ammonium formate) buffer used via reduction with Fe(II) ammonium sulfate. However, the peak at δ_H 4.87 ppm (open circle) increases from 25 min to 2880 min which likely indicates the formation of hydrated HCHO derived from the substrate. Addition of excess [^{13}C]-formaldehyde (8 mM) at 2880 min was used to confirm the chemical shift of hydrated HCHO in the mixture. The decrease in 2OG peaks at δ_H 2.93 ppm (black square) and 2.37 ppm (open square), along with the increase in the succinate peak at δ_H 2.34 ppm (black star), supports FTO mediated oxidation.

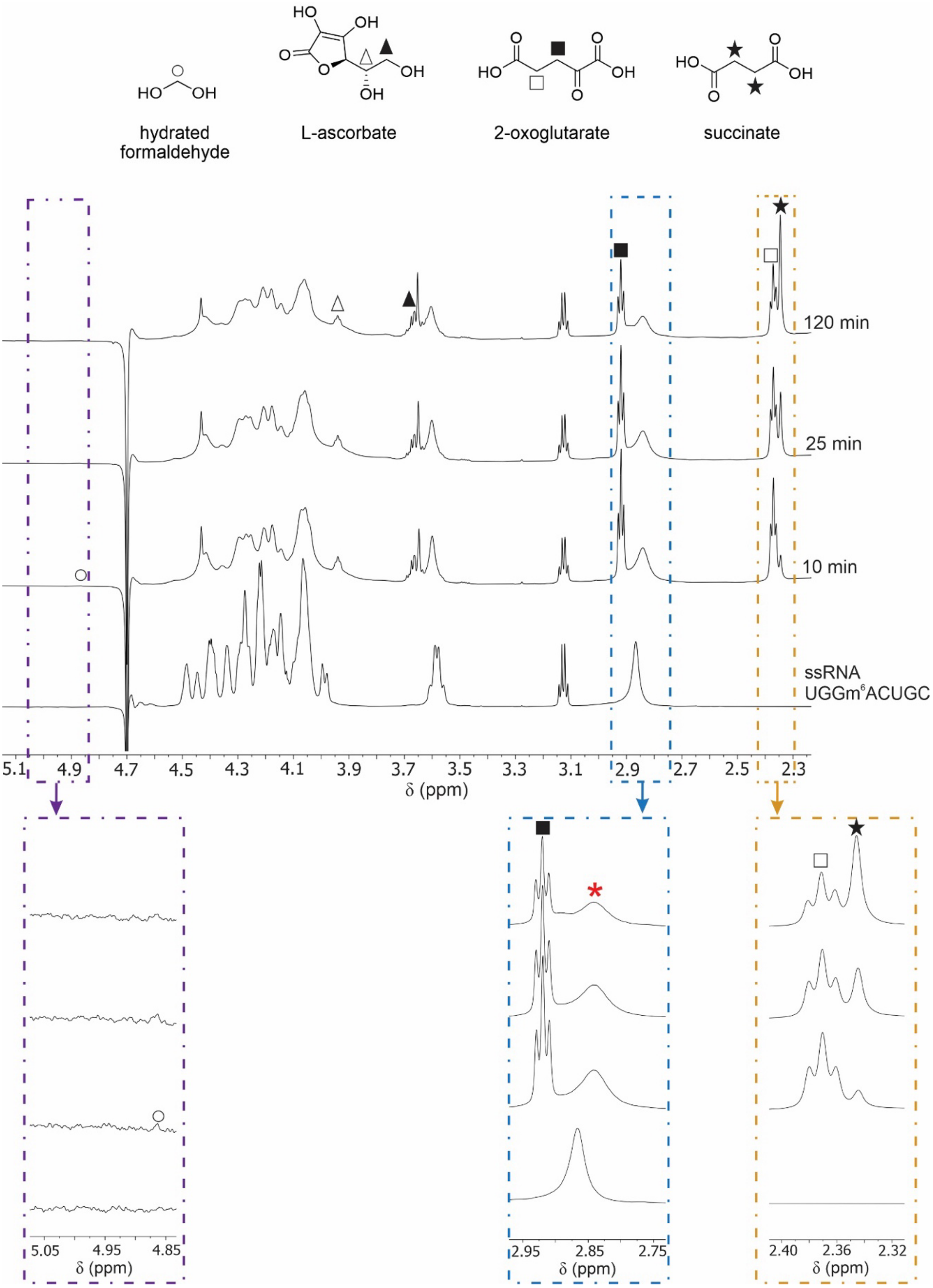

Figure S11. NMR evidence that ALKBH5 is a demethylase. ^{1}H NMR (700 MHz) time-course analysis of ssRNA UGGm^{6}ACUGC oxidation as catalysed by ALKBH5. The decrease in the N^{6}-CH_3 resonance corresponding to methyl group of ssRNA UGGm^{6}ACUGC at δ_H 2.85 ppm (red asterisk) over 120 min indicates demethylation. No evidence for a hm^{6}A hemiaminal was observed over 120 min. The decrease of 2OG peaks at δ_H 2.93 ppm (black square) and 2.37 ppm (open square), and the increase of succinate peak at δ_H 2.34 ppm (black star) over time indicate ALKBH5 mediated reaction occurred. Low levels of hydrated formaldehyde were observed at δ_H 4.87 ppm (open circle), likely derived from the (ammonium formate) buffer used via reduction involving Fe(II) ammonium sulfate.

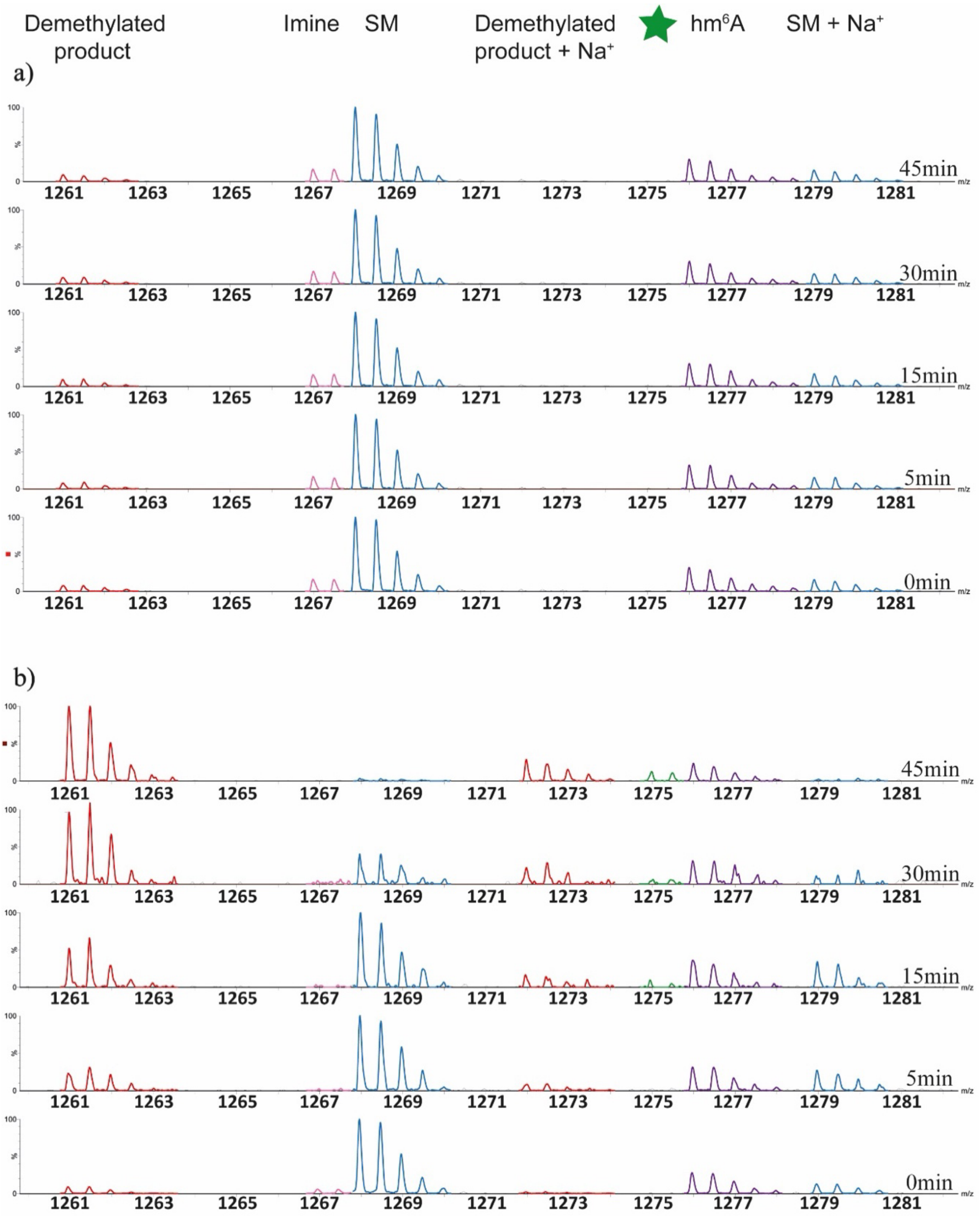

Figure S12. IP-RP-LC/ESI-MS time course analysis for the treatment of the FTO reaction products with ALKBH5. (a) FTO reaction products were generated using standard conditions; FTO was then removed, and fresh reagents (without enzyme) were added, and the mixture was analysed. The extracted ion spectra at t = 0 imply UGGm^{6}ACUGC (m/z 1268.1766, -2 charge state, blue) has been converted to hm^{6}A (m/z 1276.1832, -2, purple), which was the major product observed throughout the time-course (t = 45). Masses corresponding to low levels of the N^6-demethylated (m/z 1261.1766, -2, red) and, potentially, imine (m/z 1267.6827, -2, pink) products were observed. The relative amounts of the products were consistent up to t = 45. (b) FTO reaction products were generated using standard conditions; FTO was then removed and fresh reagents including ALKBH5 were then added and the mixture analysed (from t = 0). The results imply that the residual UGGm^{6}ACUGC (m/z 1268.1766, -2 charge state, blue) is principally converted to the N^6-demethylated product (m/z 1261.1766, -2, red); low levels of potential imine (m/z 1267.6827, -2, pink) formation were observed. Notably, levels of the demethylated product increased, while hm^{6}A (m/z 1276.1832, -2, purple) levels remained relatively constant. At t = 15, evidence for a new product, potentially f^{6}A (m/z 1274.9894, -2, green), was accrued.

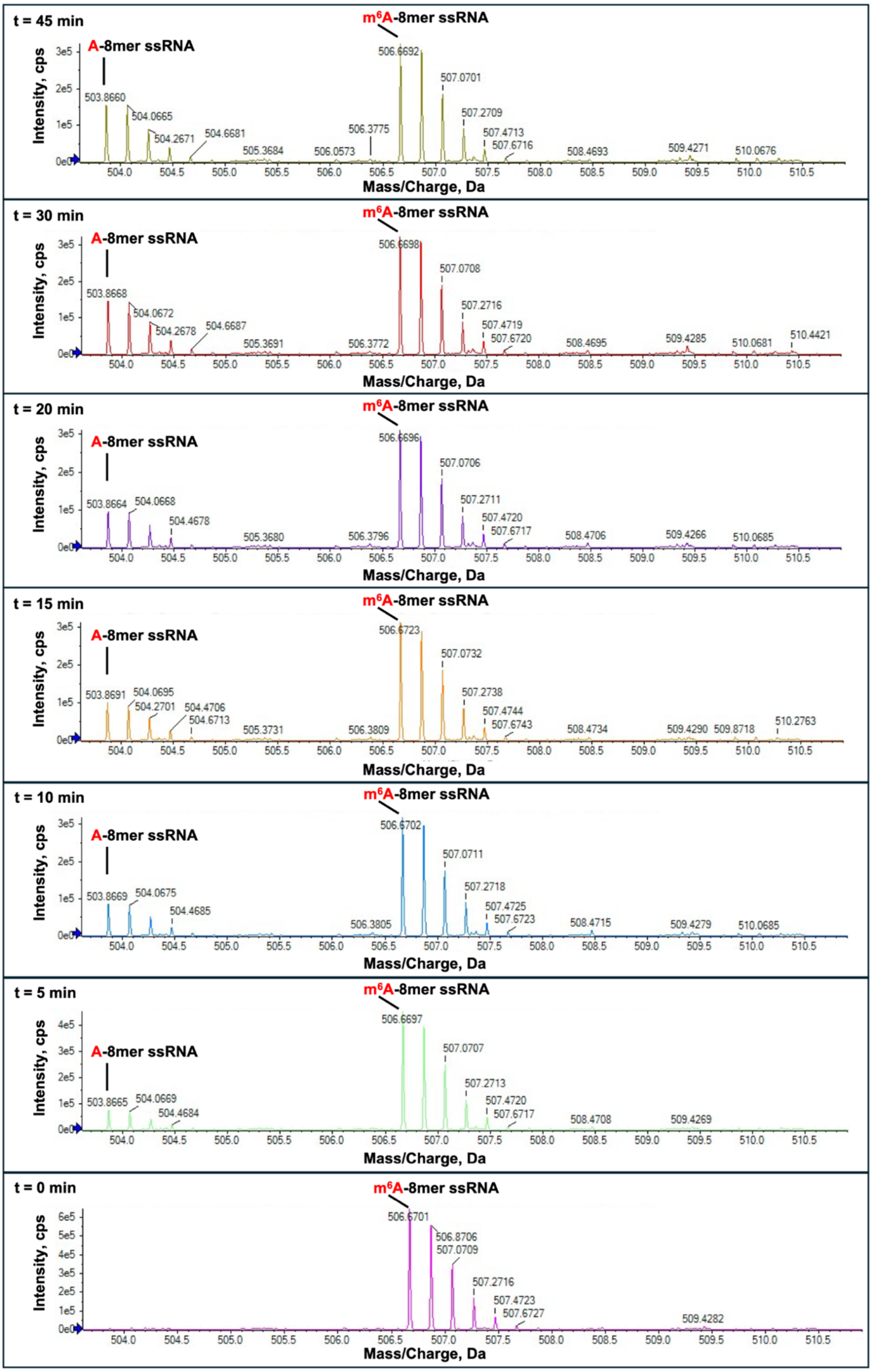

Fig. S13. QTOF-MS time course analysis for oxidation of an 8-mer ssRNA oligonucleotide with internal m^{6}A (UGGm^{6}ACUGC) as catalysed by ALKBH5$_{74-292}$. The extracted ion spectra recorded at different time points (0-45 min) indicate that the 8-mer m^{6}A ssRNA oligomer (m/z 506.67, -5) is converted to the demethylated product (m/z 503.86, -5) with no evidence for a hydroxylated product (m/z 509.86, -5) being observed.

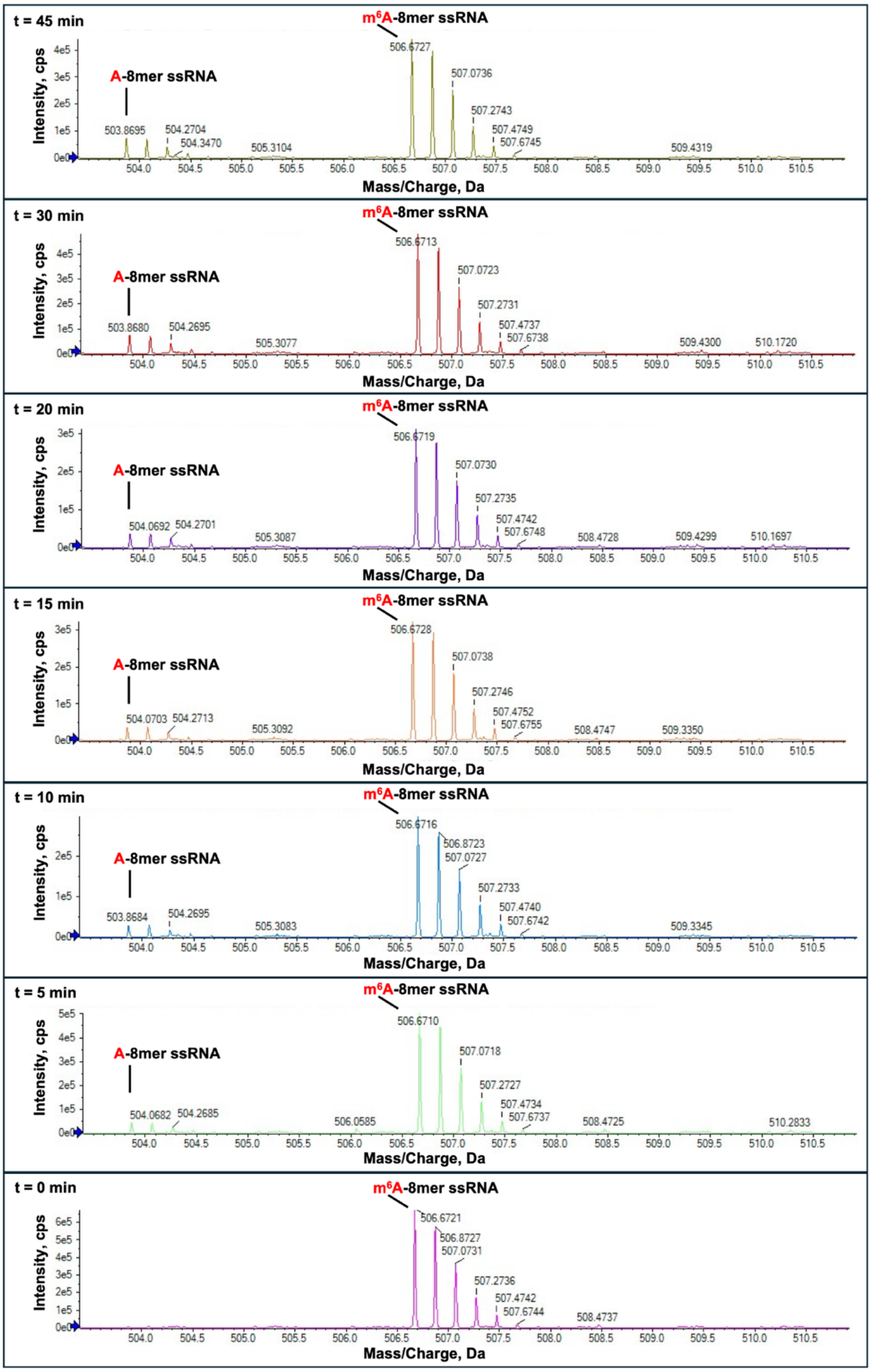

Fig. S14. QTOF-MS time course analysis for oxidation of an 8-mer ssRNA oligonucleotide with internal m^{6}A (UGGm^{6}ACUGC) as catalysed by ALKBH5$_{74\text{-}292}$ in the presence of 2,4-PDCA. The extracted ion spectra recorded at different time points (0-45 min) indicate that the 8-mer m^{6}A ssRNA oligomer (m/z 506.67, -5) is converted to the demethylated product (m/z 503.86, -5) with no evidence for a hydroxylated product (m/z 509.86, -5) being observed.

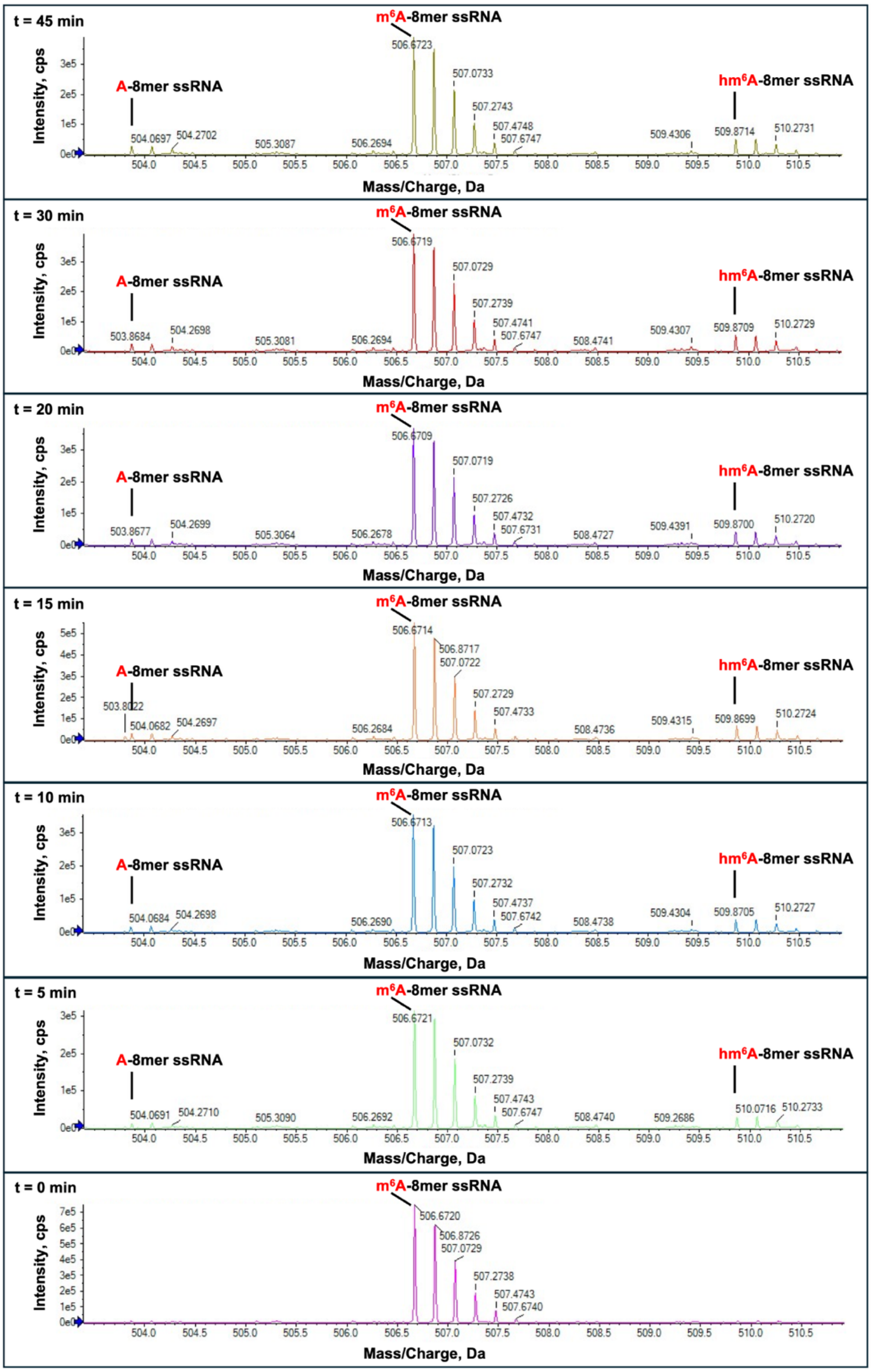

Fig. S15. QTOF-MS time course analysis for oxidation of an 8-mer ssRNA oligonucleotide with internal m^{6}A (UGGm^{6}ACUGC) as catalysed by ALKBH5$_{74\text{-}292}$ K132E. The extracted ion spectra recorded at different time points (0-45 min) indicate that the 8-mer m^{6}A ssRNA oligomer (m/z 506.67, -5) is converted to a hydroxylated product (m/z 509.86, -5), which was observed as the major product throughout the reaction time-course. The peak corresponding to the demethylated product (m/z 503.86, -5) intensifies as the reaction progresses.

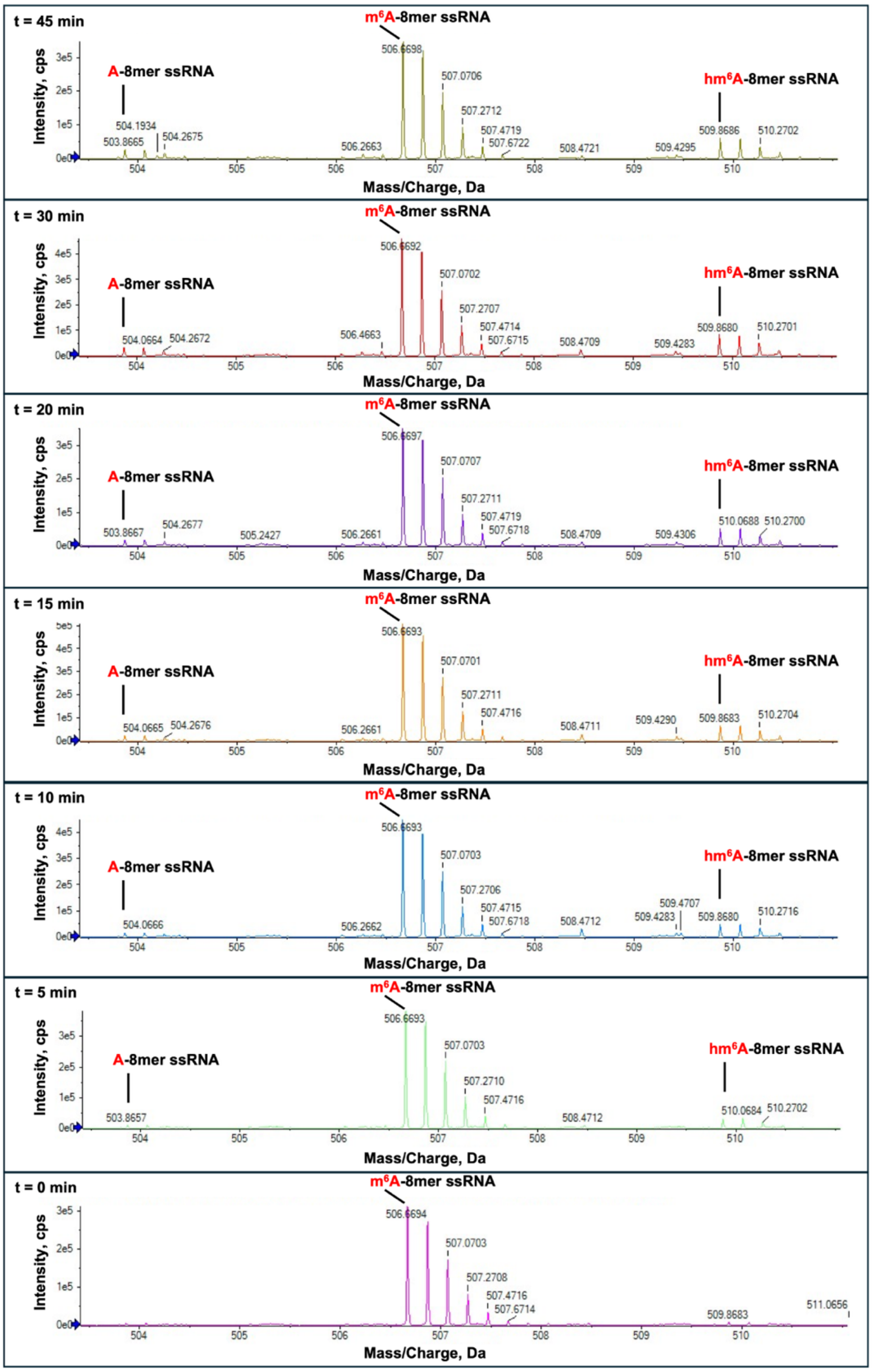

Fig. S16. QTOF-MS time course analysis for oxidation of an 8-mer ssRNA oligonucleotide with internal m^{6}A (UGGm^{6}ACUGC) as catalysed by ALKBH5$_{74\text{-}292}$ K132E in the presence of 2,4-PDCA. The extracted ion spectra recorded at different time points (0-45 min) indicate that the 8-mer m^{6}A ssRNA oligomer (m/z 506.67, -5) is converted to a hydroxylated product (m/z 509.86, -5), which was observed as the major product throughout the reaction time-course. The peak corresponding to the demethylated product (m/z 503.86, -5) intensifies as the reaction progresses.

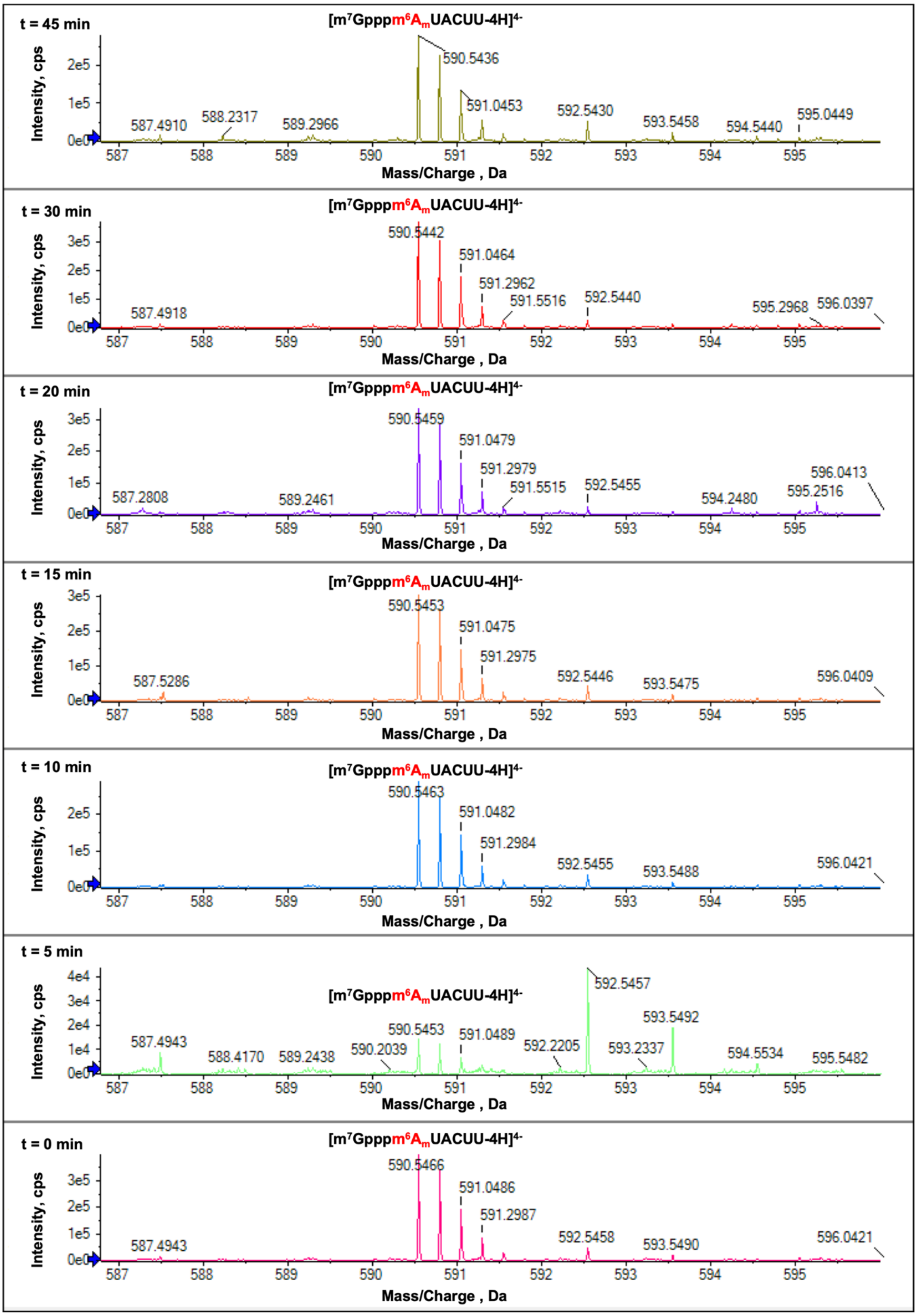

Figure S17. QTOF-MS time course analysis of 6mer m^{7}Gpppm^{6}A$_m$ ssRNA (m^{7}Gpppm^{6}A$_m$UACUU) oxidation as catalysed by ALKBH5$_{74\text{-}292}$. The extracted ion spectra recorded at different time points indicate that ALKBH5 does not oxidize the 6mer m^{7}Gpppm^{6}A$_m$ ssRNA oligo (m/z 590.54, - 4), supporting the proposal that it is a selective m^{6}A demethylase. Peaks corresponding to demethylated A$_m$ (m/z 587.06, -4), hydroxylated hm^{6}A$_m$ (m/z 594.56, -4) and f^{6}A$_m$ (m/z 594.06, -4) were not observed throughout the time course (45 min).

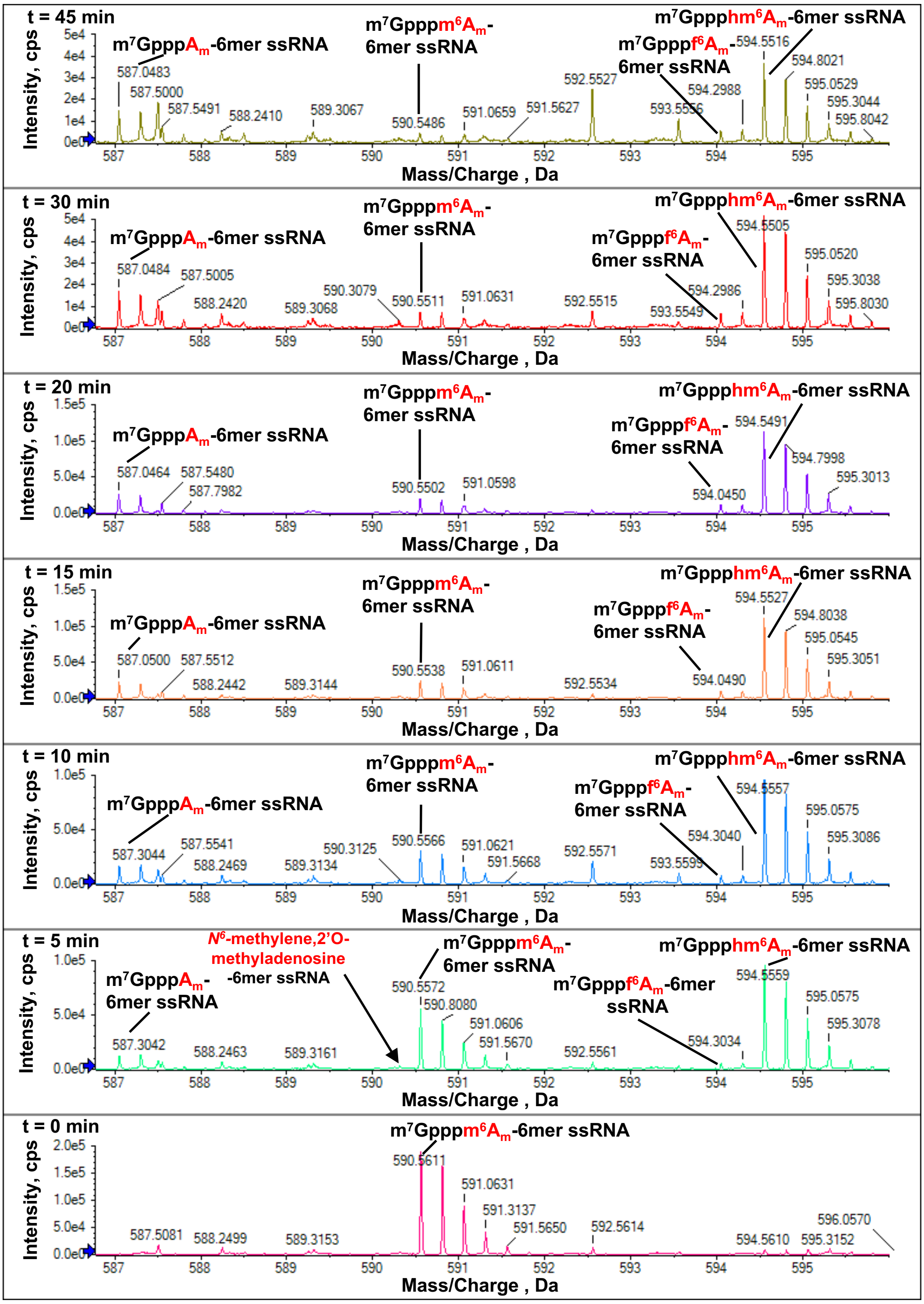

Figure S18. QTOF-MS time-course analysis for oxidation of the 6-mer m^{7}Gpppm^{6}A$_{m}$ ssRNA oligonucleotide (m^{7}Gpppm^{6}A$_{m}$UACUU) as catalysed by FTOΔ31. The extracted ion spectra recorded at different time points indicate that the 6-mer m^{7}Gpppm^{6}A$_{m}$ ssRNA oligo (m/z 590.56, - 4) is converted to a hydroxylated product (m/z 594.56, -4), which was observed as the major product throughout the time-course. The peak corresponding to a demethylated product (m/z 587.06, -4) intensifies as the reaction progresses. A peak potentially corresponding to f^{6}A$_{m}$ (m/z 594.06, -4) was observed during the reaction, albeit at low levels.

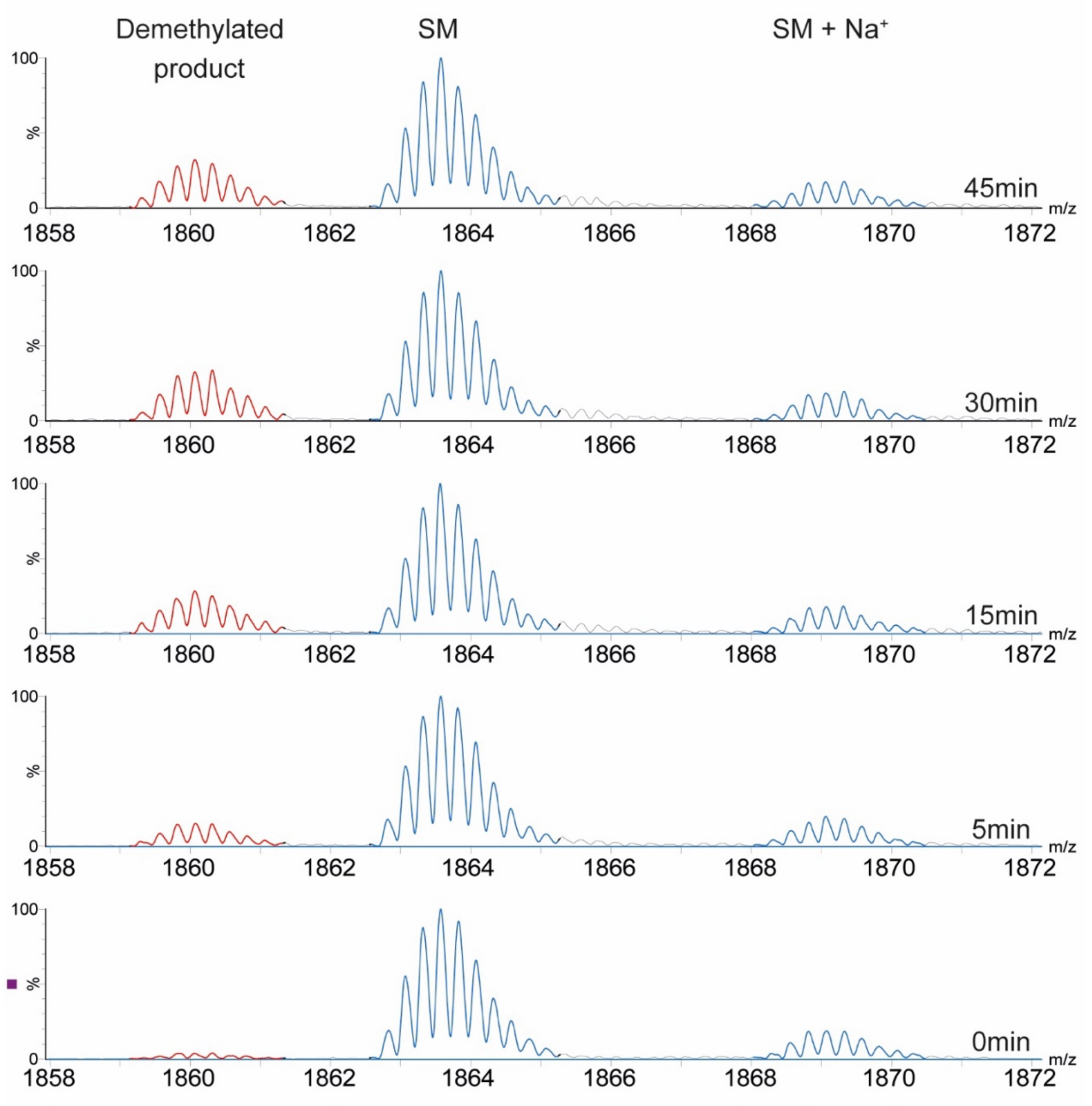

Figure S19. Evidence ALKBH2 acts as a demethylase. IP-RP-LC/ESI-MS time course analysis of ssDNA (AAAGCAGm^{1}AAATTCGAAAAAGCGAA) reaction as catalysed by ALKBH2. The extracted ion spectra indicate that AAAGCAGm^{1}AAATTCGAAAAAGCGAA (m/z 1863.5759, -4 charge state, blue) is converted to the N^1-demethylated product (m/z 1860.0623, -4, red), which was the only product observed throughout the time-course (45 min). No evidence for hm^{6}A, imine or f^{6}A formation was accrued under the tested conditions. SM: starting material.

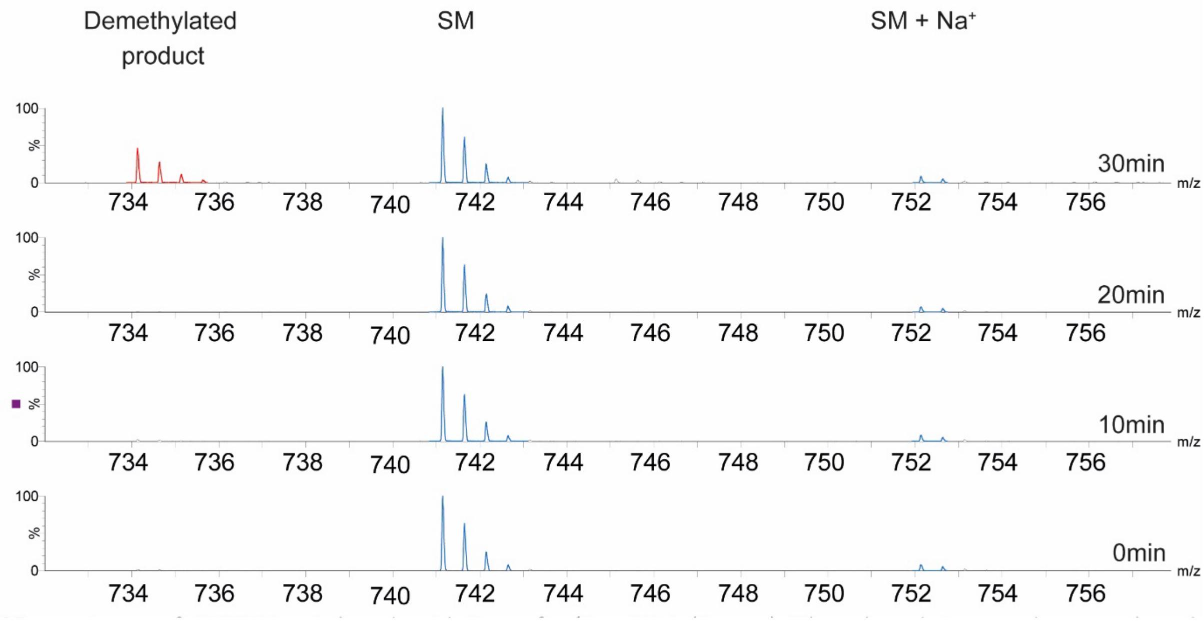

Figure S20. Evidence ALKBH3 acts as a demethylase. IP-RP-LC/ESI-MS time course analysis of ssDNA CAm^1AAT reaction as catalysed by ALKBH3. The extracted ion spectra indicate that CAm^1AAT (m/z 741.1453, -2 charge state, blue) is converted to a N^1-demethylated product (m/z 734.1332, -2, red), which was the only observed product throughout the time-course (30 min). No evidence for hm^6A, imine, or f^6A formation was accrued under the tested conditions. SM: starting material.

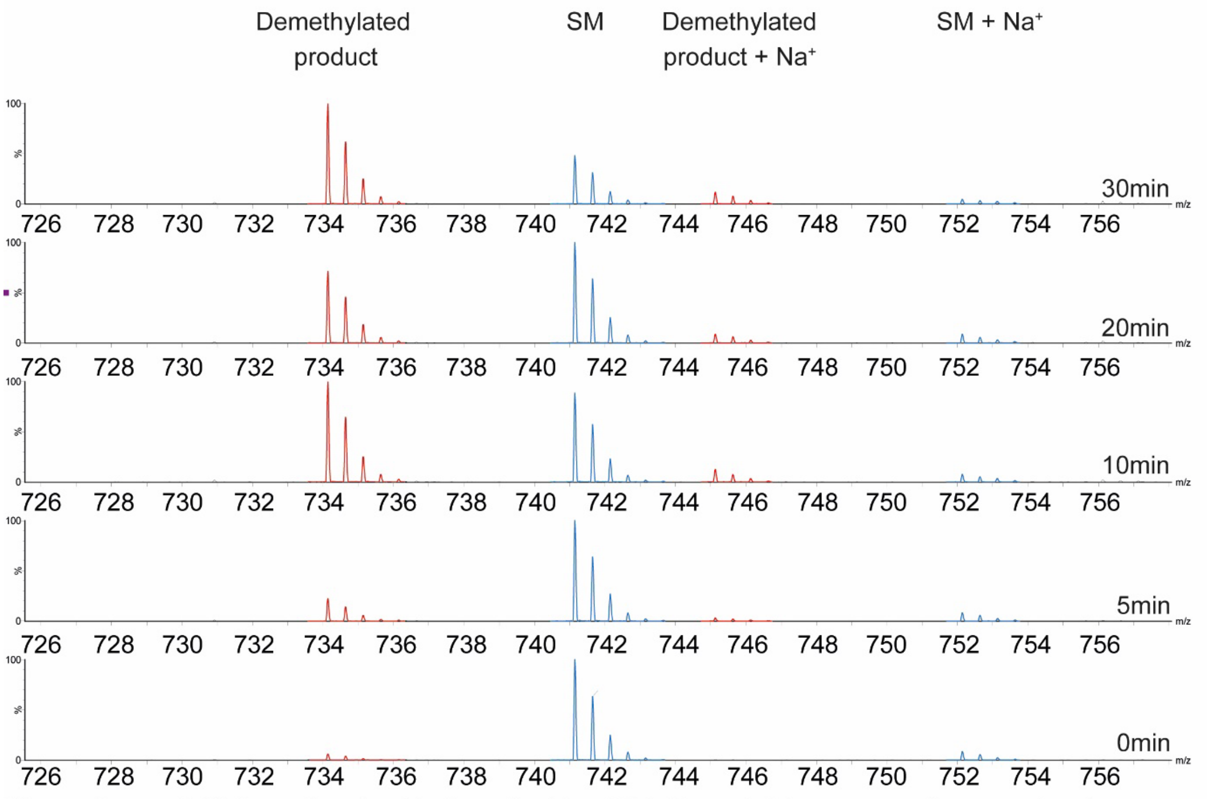

Figure S21. Evidence AlkB acts as a demethylase. IP-RP-LC/ESI-MS time course analysis of ssDNA CAm^{1}AAT oxidation as catalysed by AlkB. The extracted ion spectra indicate that CAm^{1}AAT (m/z 741.1453, -2 charge state, blue) is converted to the *N^1*-demethylated product (m/z 734.1388, -2, red), which was the only observed product throughout the time-course (30 min). No evidence for hm^{6}A, imine, or f^{6}A formation was accrued under the tested conditions. SM: starting material.

Supplementary Reference

50. Shishodia, S., Zhang, D., El-Sagheer, A., Brown, T., Claridge, T., Schofield, C. and Hopkinson, R. (2018) NMR analyses on N-hydroxymethylated nucleobases–implications for formaldehyde toxicity and nucleic acid demethylases. *Org Bio Chem*, **16**, 4021-4032.
